# Development of Blueberry-Derived Extracellular Nanovesicles for Immunomodulatory Therapy

**DOI:** 10.3390/pharmaceutics15082115

**Published:** 2023-08-10

**Authors:** Tuong Ngoc-Gia Nguyen, Cuong Viet Pham, Rocky Chowdhury, Shweta Patel, Satendra Kumar Jaysawal, Yingchun Hou, Huo Xu, Lee Jia, Andrew Duan, Phuong Ha-Lien Tran, Wei Duan

**Affiliations:** 1 School of Medicine, Faculty of Health, Deakin University, Geelong Waurn Ponds Campus, Geelong, VIC 3216, Australia; nnguyen9@nymc.edu (T.N.-G.N.); cuong.pham@baker.edu.au (C.V.P.); chowdhuryro@deakin.edu.au (R.C.); patelshw@deakin.edu.au (S.P.); sjaysawal@deakin.edu.au (S.K.J.); 2 Laboratory of Tumor Molecular and Cellular Biology, College of Life Sciences, Shaanxi Normal University, 620 West Chang’an Avenue, Xi’an 710119, China; ychhou@snnu.edu.cn; 3 College of Materials and Chemical Engineering, Minjiang University, Fuzhou 350108, China; 2627@mju.edu.cn (H.X.); leejia2000@mju.edu.cn (L.J.); 4 School of Medicine, Faculty of Medicine, Nursing and Health Sciences, Monash University, Clayton, VIC 3800, Australia; adua0001@student.monash.edu

**Keywords:** plant EV, biomarker, pathogen-related proteins, class I chitinase, drug delivery, immunomodulatory

## Abstract

Over the past decade, there has been a significant expansion in the development of plant-derived extracellular nanovesicles (EVs) as an effective drug delivery system for precision therapy. However, the lack of effective methods for the isolation and characterization of plant EVs hampers progress in the field. To solve a challenge related to systemic separation and characterization in the plant-derived EV field, herein, we report the development of a simple 3D inner filter-based method that allows the extraction of apoplastic fluid (AF) from blueberry, facilitating EV isolation as well as effective downstream applications. Class I chitinase (PR-3) was found in blueberry-derived EVs (BENVs). As Class I chitinase is expressed in a wide range of plants, it could serve as a universal marker for plant-derived EVs. Significantly, the BENVs exhibit not only higher drug loading capacity than that reported for other EVs but also possess the ability to modulate the release of the proinflammatory cytokine IL-8 and total glutathione in response to oxidative stress. Therefore, the BENV is a promising edible multifunctional nano-bio-platform for future immunomodulatory therapies.

## 1. Introduction

Plant-derived extracellular vesicles (EVs) have emerged as an unconventional means of self-protection from invasion by pathogens [1]. In addition, plant-derived EVs can block fungal growth by generating papillae and encasements using cargos contained in the EVs, namely antifungal peptides and small RNAs [1]. For the pharmaceutical industry, plant-derived EVs are an attractive alternative to mammalian-derived EVs due to their human-friendly characteristics such as their non-immunogenic properties [2]. 

Concerns have been raised regarding the safety of mammalian-derived EVs since they might transfer hazardous materials such as zoonotic or human pathogens [2] or pro-cancerous elements [3] from the parental cancer or immortalized cells to the recipient human cells. In contrast, plant-derived EVs are less immunogenic, contain intrinsic therapeutic abilities, display better cellular uptake, and are able to withstand the harsh environment of the gastrointestinal tract [2]. Furthermore, the preparation of plant-derived EVs is less complex than that of animal-derived EV platforms [2]. In addition, plant-derived EVs provide better protection of their cargoes, securely concealing the cargoes from proteinases and nucleases to minimize enzymatic decomposition [4]. Therefore, the development of plant-derived EVs as effective therapeutic and delivery platforms provides a new weapon to the armamentarium for cancer therapeutics, including cancer immunotherapy. However, compared to their mammalian EV counterparts, the field of plant-derived EVs faces a series of technical challenges, including techniques for isolation and characterization, the ability to carry a high quantity of payload, and the evaluation of internalization into and interaction with targeted cells [2].

Currently, plant-derived EV-like nanoparticles are commonly isolated from juices generated by grinding or squeezing methods. The collected plant-derived EVs from these methods do not come from the extracellular space only, but are contaminated by microsomal fragments during the grinding and juicing processes [5]. To protect the plasma membrane from collapsing and to retain cell wall-bound proteins, several approaches have been introduced such as vacuum infiltration–centrifugation [6,7,8,9], pressure dehydration with careful temperature adjustment [10,11], filtration using appropriate simulated apoplastic solutions [12], vacuum perfusion [13], and elution [14]. Nevertheless, these techniques have some limitations. For example, the elution method is unable to discriminate between apoplastic fluids and protoplasts, resulting in more than 30% contamination from the protoplast fluids present in the collected solution. Moreover, the ratio between the cutting surface and tissue volume determines the level of contamination of released intracellular solutes [10]. On the other hand, the vacuum perfusion and pressure dehydration techniques require complicated and cumbersome instruments, rendering them unsuitable for large-scale production. Although perfusion and infiltration methods are fast and inexpensive, there is a possibility of altering the ionic composition, pH value, and metabolite concentration of apoplastic fluid due to additive-stimulated apoplastic solutions or water infiltration [15]. In addition, it is impractical to evaluate the physiological concentration of distinct metabolites and molecules in the apoplastic fluid ex situ [15]. Finally, the vacuum infiltration–centrifugation method generates a low amount of apoplastic proteins in waxy-coated leaves (e.g., rice and maize), hence reducing the extracted proteome [16]. Therefore, there is an urgent unmet pharmaceutical need for effective methods to extract plant-derived EVs from extracellular fluid outside the plasma membrane to achieve high-quality plant-derived EVs for clinical therapy.

Another challenge that impedes the development of plant-derived EVs is the lack of suitable and reliable biomarkers for plant-derived EVs. The development of plant EV biomarkers will contribute to the advancement of all aspects in the field, including isolation and characterization techniques, classifications, and downstream applications. So far, only a few plant-derived EV proteomes from apoplasts have been analyzed, such as those from olive pollen grains [17], *A. thaliana* (thale cress) leaves [18,19], sunflower seedlings [20], and *N. benthamiana* leaves [18]. In addition, no commercial antibodies against biomarkers for plant-derived EVs are available, hindering further progress in the field. Pinedo et al. suggested that there are three possible EV markers that were presented in both plant and mammalian proteomes, including heat shock protein 70 (HSP70), S-adenosyl-homocysteinase, and glyceraldehyde 3 phosphate dehydrogenase (GAPDH) [21]. Other possible cytosolic proteins in plant-derived EVs include native Pattelin-1 and -2 (PATL-1 and PATL-2), which participate in membrane-trafficking activities [21]. Unfortunately, these protein families are also found in plant-derived nanoparticles that might have been contaminated by destructive processes, together with proteins originating from plant-derived EVs such as glutathione S-transferase and annexin [22,23,24,25]. Pinedo et al. also suggested that plant-derived EV biomarkers should persist across different plant species and accumulate at high levels in EVs instead of in whole cells [21]. However, the diversity of plant proteomes poses a major challenge for the development of biomarkers for plant-derived EVs. 

In this study, we aimed to address several challenges in the development of plant-derived EVs. Firstly, we established a comprehensive EV isolation system for succulent fruits using blueberry (BB) as a model plant source, followed by extensive characterization and an attempt to identify specific biomarkers for blueberry-derived EVs (BENVs) as well as plant-derived EVs in general. We have identified a general biomarker for plant-derived EVs from different edible fruits and showed that BENVs have a much higher drug loading capacity than other types of EVs. The confirmation of the presence of anthocyanins in blueberry-derived EVs supports their potential health benefits, such as antioxidant properties, protection against cardiovascular diseases, cancers, diabetes, and UV-B radiation [26]. Our results suggest that the isolated BENVs retain the antioxidant properties of the whole fruit and are thus a promising next generation of edible drug delivery vehicle with intrinsic anti-inflammatory capacity. 

## 2. Materials and Methods

### 2.1. Materials

Aspirin (Sigma-Aldrich, St. Louis, MO, USA, Cat #5376) and curcumin (Sigma-Aldrich, Cat #C1386) were purchased from Sigma Aldrich (USA). The solvents were used at HPLC grade and other analytical grade chemicals were utilized without further purification.

### 2.2. Cell Culture

Human colon adenocarcinoma epithelial cell line (Caco-2) and human colorectal adenocarcinoma cell line (HT-29) were acquired from American Type Culture Collection (ATCC) (Manassas, VA, USA). All cells were grown in Dulbecco’s modified Eagle’s medium (DMEM, Invitrogen, Waltham, MA, USA, Cat #12800-017) supplemented with 12–15% fetal bovine serum (FBS, Hyclone, Logan, Utah, Cat #A50111-5039), 100 U/mL penicillin and 100 μg/mL streptomycin (Invitrogen^TM^, Cat #15070-063). Cells were kept at 37 °C and 5% CO_2_ in an incubator.

### 2.3. Preparation of Plant-Derived Nanovesicles

Blueberries were directly purchased from Tuckerberry Hill Blueberry Farm (Drysdale Victoria, Australia), or from Coles Supermarket (Waurn Ponds, VIC, Australia). Blueberries were washed 3 times with Milli-Q water. The unripe and rotten fruits were eliminated. After that, the blueberries were frozen and stored at −20 °C for further use.

Apoplastic fluid (AF) was extracted from blueberries using the centrifugation method modified from a study by Wada et al. [27]. Briefly, each blueberry was cut with a disposable blade and 2 mm of the pericarp from the stylar (distal) was removed as the residual part. The flat cut surface was immediately placed onto a homemade, reusable filter tube produced by 3D printing technique (Figure S1). Subsequently, the filter tube was put into the 50 mL conical tube. Next, the tube was centrifuged at 30× *g* for 10 min at 4 °C and the solution at the bottom of the tube was discarded to eliminate symplast contamination from the cutting method and damaged cells [28]. The filter tube containing blueberry was transferred into a clean 50 mL conical tube and then centrifuged at 200× *g*, 4 °C for another 10 min to collect the apoplastic fluid. The apoplastic fluid was filtered through a 0.8 μm mixed cellulose esters membrane to remove cellular debris and unwanted organelles before being stored at −20 °C for further use.

In this study, BENVs were isolated from AF using differential centrifugation approaches modified from a method described by Regente et al. [20]. Briefly, 13 mL of AF was centrifuged at 2000× *g* at 4 °C for 20 min and then 10,000× *g* for 30 min at 4 °C to eliminate cells and cellular debris in the pellet. The supernatants were collected for EV collection using differential ultracentrifugation. Minced juices (MJs) of different fruits were prepared as follows: Blueberry juice was produced by homogenizing frozen blueberries in a blender for 5 min to collect juice; grapefruits with skin removed were cut in half before squeezing in a cold room to collect juice. In the case of grapes, after removal of the skin, they were homogenized in a blender for 2 min. The grape juice was diluted with cold PBS (1:1) and centrifuged at 2000× *g* for 10 min at 4 °C to collect the supernatant. The collected juices were filtered through a 0.45 µm mixed cellulose esters syringe filter. Subsequently, all filtered juices were centrifuged at 2000× *g* for 20 min followed by centrifuged at 10,000× *g* for 30 min to eliminate cells and cellular debris in the pellets. Finally, the supernatants collected from AF and juices were centrifuged at 40,000× *g* and 100,000× *g*, respectively, for 6 h at 4 °C using the Beckman Coulter Optima L-90K Ultracentrifuge, and the pellets were suspended in 400 µL PBS.

### 2.4. Attenuated Total Reflection Fourier-Transform Infrared Spectroscopy (ATR-FTIR)

Solution samples were directly examined on the “Gold Gate” single reflection diamond ATR accessory using a Bruker Vertex 70 FTIR spectrometer at a resolution of 4 cm^−1^. The instrument was performed with an average of 32 scans and in the wavelength range of 600–4000 cm^−1^. The FTIR data was analyzed as described by Mihály et al. [29].

### 2.5. Nanoparticle Tracking Analysis (NTA)

NTA analysis was performed as described in our previous study [30]. In brief, 1 mL of diluted sample was loaded into the chamber of NTA equipment, and three videos were recorded in 60 s. The average size and number of particles from three recorded videos were then determined.

### 2.6. Transmission Electron Microscopy (TEM)

Ten microliters of BENVs collected at the centrifugal force of 40,000× *g* or 100,000× *g* was fixed in 50 µL of 2% paraformaldehyde (Sigma-Aldrich). Then, 5 µL of the mixed solution was transferred onto a Formvar carbon-coated electron microscopy grid and incubated for 20 min in a closed petri dish. The grid was subsequently washed by 100 µL of PBS for 2 min and transferred with 50 µL of 1% glutaraldehyde for 5 min, followed by washing with milli-Q water for 2 min. Next, the grid was negatively stained with 2% phosphotungstic acid (Sigma-Aldrich) for 2 min, and the excess staining solution was removed by a piece of filter paper before allowing to dry for 15 min in air. The TEM images were obtained using JEOL JEM 2100 TEM at 100 kV.

### 2.7. Protein Extraction of Plant Samples

Plant proteins were extracted using Kikuchi’s method with minor modification [31]. Briefly, one mL of sample (containing either 1.8 mg/mL or 0.9 mg/mL of total protein) was mixed with 10 mL of extraction buffer (8 M urea, 50 mM Tris–HCl, pH 7.6, 2% (*w*/*v*) Triton X-100, 5 mM EDTA, 10 mM dithiothreitol (DTT) and protease inhibitor mixture tablet (Roche, Basel, Switzerland, Cat #11697498001)) for 5 min at 25 °C. The mixture was centrifuged at 16,000× *g*, for 10 min at 4 °C. Proteins in the supernatants were precipitated by adding 40 mL of acetone and incubation at −20 °C overnight. The suspension was centrifuged at 2300× *g* for 5 min at 4 °C. The pellets were washed twice with 75% acetone and dissolved in protein extract solution (1.2% (*w*/*v*) sodium dodecyl sulfate (SDS), 50 mM Tris-HCl, pH 6.8, 1% (*v*/*v*) β-mercaptoethanol, 20% glycine, and 0.001% (*w*/*v*) bromophenol blue), at 95 °C for 5 min. Insoluble proteins were eliminated by a brief centrifugation at 10,000× *g* for 1 min at 4 °C. The final protein concentration in samples were determined using BCA protein assay kit (Thermo Scientific, Waltham, MA, USA) and stored at −80 °C for further analysis.

### 2.8. Aptamer Binding Assay Using Fluorescence Polarization

Modified SYL3C aptamer labelled with FAM (FAM-anti-EpCAM-Chol aptamer) [32] (25 nM) was synthesized from IDT. The aptamers were folded by denaturing at 85 °C for 5 min in binding buffer (PBS containing 5 mM of MgCl_2_), followed by returning to room temperature over 10 min and a refolding at 37 °C for at least 15 min.

BENVs were lysed by incubating isolated BENVs with 0.05% Triton X-100 for 30 min at room temperature. An equal volume of intact BENVs or lysed BENVs (1.56 × 10^8^ particles/mL) was added into a 96-well black plate and mixed with 5 nM of modified SYL3C aptamer in PBS at different ratios to obtain a total reaction volume of 100 µL. The plate was placed onto an orbital shaking incubator for 15 or 30 min at room temperature in the dark, with gentle shaking at 30 rpm. Subsequently, the fluorescence polarization values were measured with an excitation at 485 nm and an emission at 528 nm using VICTORTM X5 Plate Reader.

### 2.9. DiD-Labeled Aptamer-Conjugated BENVs

The labeling of aptamer-conjugated BENVs (Apt-BENVs) with Vybrant DiD solution (Thermo Fisher, Waltham, MA, USA, Cat #V22887) was performed as follows: 1 mL of aptamer-conjugated BENVs (1.56 × 10^7^ particles/mL) was incubated with 6 µL of Vybrant DiD solution for 30 min, followed by two washes with 1 mL of PBS followed by ultracentrifugation at 4 °C, 100,000× *g* for 1 h each to remove free dyes. The pellet was resuspended in filtered PBS and stored at −80 °C for further analysis.

### 2.10. Cellular Uptake

Caco-2 cells and HT-29 cells were seeded on 8-chamber slides (Thermo Fisher, Cat #154534PK) at 4000 cells/well and 3000 cells/well, respectively. After culturing for 48 h at 37 °C, the cells were washed twice with Hank’s balanced salt solution (HBSS, Sigma-Aldrich). Then, 200 µL of fresh culture media containing DiD-BENVs-Apt (3 × 10^6^ particles/mL) was added to each well and incubated for 6 h at 37 °C. Prior to analyzing by microscope, cells were extensively washed with 0.5 mL of HBSS for three times to remove the non-internalizing DiD-BENVs-Apt. Next, cells were treated with 50 µL of nucleic indicator Hoechst 33342 (2 μg/mL) and incubated at room temperature in the dark for 10 min. Subsequently, cells were washed extensively with HBSS to remove the DiD-BENVs-Apt bound on the cell surface. The uptake of DiD-BENVs-Apt was analyzed using ECLIPSE Ti2 inverted microscope and flow cytometry.

### 2.11. Transport Study

Caco-2 cells were seeded onto Corning Transwell inserts (0.4 µm pore diameter, 1.12 cm^2^) for 21 days prior to studying of drug transportation. Before analysis, the inserts were washed twice and equilibrated with transport medium (i.e., 25 mM HEPES in HBSS (pH 7.4)). An addition of 1% DMSO in the transport buffer served as co-solvent. The integrity of the Caco-2 cells monolayer and their differentiation were evaluated by measuring the trans-epithelial electrical resistance (TEER) using the Millicell^®^ ERS-2. The resistance value is presented as Ω (electric resistance), and the TEER value was calculated as:

TEER (Ω·cm^2^) = (Ω_cell insert_ − Ω_cell-free insert_) × 1.12 cm^2^

Next, 0.5 mL of DiD-labeled BENVs (10^8^ particles/mL) in transport buffer were added on the apical side of the chamber and 1.5 mL of transport buffer was added on the basolateral side. After incubation in an orbital shaking incubator at 37 °C, 150 µL of media was collected from the basolateral side at different intervals of incubation (i.e., 0, 1, and 3 h), and fresh media was added to maintain the volume at the lower chamber. At the end of the experiment, the TEER was measured again to examine the intact BENVs of the Caco-2 cell monolayer. Moreover, the transported DiD-BENVs in the basolateral chamber were determined by fluorescence microscopy.

### 2.12. Preparation of Drug-Loaded BENVs

Drug-loaded BENVs were prepared by conventional incubation method. BENVs at different concentrations (i.e., 500 µg/mL or 1000 µg/mL of total proteins) after thawing were immediately kept at different temperatures (i.e., 4 °C or 25 °C) for 5 min or 10 min to determine the effects of conditions before drug loading. Next, payload, either 120 µM aspirin or 100 µM curcumin in absolute ethanol, was incubated with the designed amounts of BENVs at different temperature (i.e., 25 °C and 37 °C), shaking condition (i.e., 150 rpm and 200 rpm), and incubation time (i.e., 15, 20, 30, 45, and 60 min). All the experiments with curcumin were performed in the dark. After the incubation, the mixture was centrifuged at a low speed of 5000× *g* for 10 min at 4 °C to remove the unbound precipitated drugs. Supernatants were collected and designated as W1 samples, and the pellets were suspended in 1 mL of filtered PBS. Subsequently, drug-loaded BENVs were washed twice in filtered PBS solution by a centrifugation at 100,000× *g* for 45 min at 4 °C. The collected supernatants were designated as W2 and W3 for unbound drugs in the solution and on the BENV surface, respectively. The final pellets were resuspended in 200 µL of filtered PBS and stored at −20 °C for further experiments. Drug concentrations were determined by HPLC [33]. The percentage of drug loading and entrapment efficiency were calculated as follow:

$$\mathrm{Drug}\;\mathrm{loading}\;\left(\%\right)=\frac{\mathrm{Amount}\;\mathrm{of}\;\mathrm{drug}\;\mathrm{in}\;\mathrm{drug}-\mathrm{loaded}\;\mathrm{BENVs}}{\mathrm{Amount}\;\mathrm{of}\;\mathrm{exosomal}\;\mathrm{proteins}\;\mathrm{in}\;\mathrm{drug}-\mathrm{loaded}\;\mathrm{BENVs}}\times 100$$

$$\mathrm{Entrapment}\;\mathrm{efficiency}\;\left(\%\right)=\frac{\left(\mathrm{Drug}\;\mathrm{added}-W1-W2-W3\right)}{\mathrm{Drug}\;\mathrm{added}}\times 100$$

### 2.13. Cell Viability Assay

MTT assay was used to determine cell viability. Caco-2 cells were seeded at 5000 cells/well in a 96-well plate and incubated at 37 °C and 5% CO_2_ in an incubator for 24 h. Then, the medium was removed, and cells were treated with 100 µL of fresh culture medium containing either free curcumin, BENVs, or curcumin-loaded BENVs at various concentrations, followed by an incubation for 48 or 72 h at 37 °C. Non-treatment cells and culture medium only served as the negative controls and were designated as cells without test compound and medium control, respectively. Next, 20 µL of MTT in PBS (5 mg/mL) was added into each well and incubated for another 4 h at 37 °C, followed by the addition of 150 µL of DMSO to solubilize MTT. The absorbance was measured at 570 nm by a VICTOR TM X5 Plate Reader. The viability of cells was calculated as follows:

$$\mathrm{Viability}\;\left(\%\right)=\left(\frac{{\overset{—}{M}}_{\mathrm{treated}\;\mathrm{cells}}-{\overset{—}{M}}_{\mathrm{medium}\;\mathrm{control}}\;}{{\overset{—}{M}}_{\mathrm{control}\;\mathrm{cells}\;\mathrm{without}\;\mathrm{test}\;\mathrm{compound}}-{\overset{—}{M}}_{\mathrm{medium}\;\mathrm{control}}}\right)\times 100$$

### 2.14. Evaluation of BENVs’ Capability in Immune Modulation

The capability in modulating immune systems was evaluated based on the release of pro-inflammatory cytokines IL-8 in Caco-2 cells during hydrogen peroxide (H_2_O_2_)-induced oxidative stress. IL-8 release in supernatants were measured by a Human IL-8/CXCL8 ELISA Kit as per the manufacture’s instruction (Sigma-Aldrich, Cat #RAB0319-1KT). Total glutathione was measured to evaluate the intrinsic cellular antioxidant responses during the anthocyanin-rich treatments [34]. Post-treatment Caco-2 cells were washed with cold PBS three times and immersed in cold PBS before being collected by scraping method using Falcon™ Cell Scrapers. The collected samples were centrifuged at 600× *g* for 5 min at 4 °C and the pellets were suspended in 500 µL of ice cold 5% aqueous 5-sulfosalicylic acid dihydrate (SSA). Cells were disrupted by sonicating in ice-water bath for 5 min, followed by an incubation at 4 °C for 10 min. Subsequently, samples were centrifuged at 14,000× *g* for 10 min at 4 °C, and the supernatants were collected and stored at −80 °C for further analysis. The total GSH was measured by a Glutathione Fluorescent Detection Kit (Invitrogen, Cat #EIAGSHF) following the manufacturer’s instructions.

### 2.15. Immunoblotting Analysis

The collected plant proteins were separated on SDS-PAGE gel and blotted onto nitrocellulose membranes (Whatman, Maidstone, UK, Cat. 10401196). Goat anti-rabbit HRP-conjugated secondary antibody (Thermo Fisher Scientific, Waltham, MA, USA; Cat #31460) was used to detect PR-3/CHN|Class I chitinase (Agrisera, Vännäs, Sweden, Cat #AS07 207) and PR-2|GLU I|Class I beta-1,3-glucanase (Agrisena, Vännäs, Sweden, Cat #AS07 208).

### 2.16. Statistical Analysis

All samples were prepared in triplicate and results were expressed as means ± standard deviations unless otherwise stated. Statistical analysis was executed by GraphPad PRISM 8 with one-way analysis of variance (ANOVA) for differences among multiple groups and two-sided paired Student’s *t*-test for differences between two specific groups. A *p* value ≤ 0.05 was considered statically significant.

## 3. Results

### 3.1. Preparation and Characterization of Blueberry-Derived Extracellular Nanovesicles (BENVs)

In 1990, Welbaum and Meinzer et al. proposed a method to extract apoplastic fluid (AF) from sugarcane using serial low-speed centrifugation accompanied with a 0.8 µm cellulose acetate filter in a 5 mL tube [28]. However, this method can only be applied to watery plants because the viscosity sap will block the filter as soon as they are in contact. In addition, low-speed centrifugation is unable to force the high viscosity AF through the filter. Therefore, we introduced a new strategy for the preparation of BENVs in which a tube holder with small holes (0.5–1.5 mm) separated the AF out of the fruit at low-speed centrifugation. Subsequently, the collected AF was filtered through a 0.8 μm mixed cellulose esters membrane using a vacuum filter apparatus to remove cellular debris and unwanted organelles. The strategy of serial low-speed centrifugation allows AF to be collected at its purest form as the symplastic contaminations from the cutting process and damaged cells are eliminated [28] (Figure S1). 

**Figure 1:**
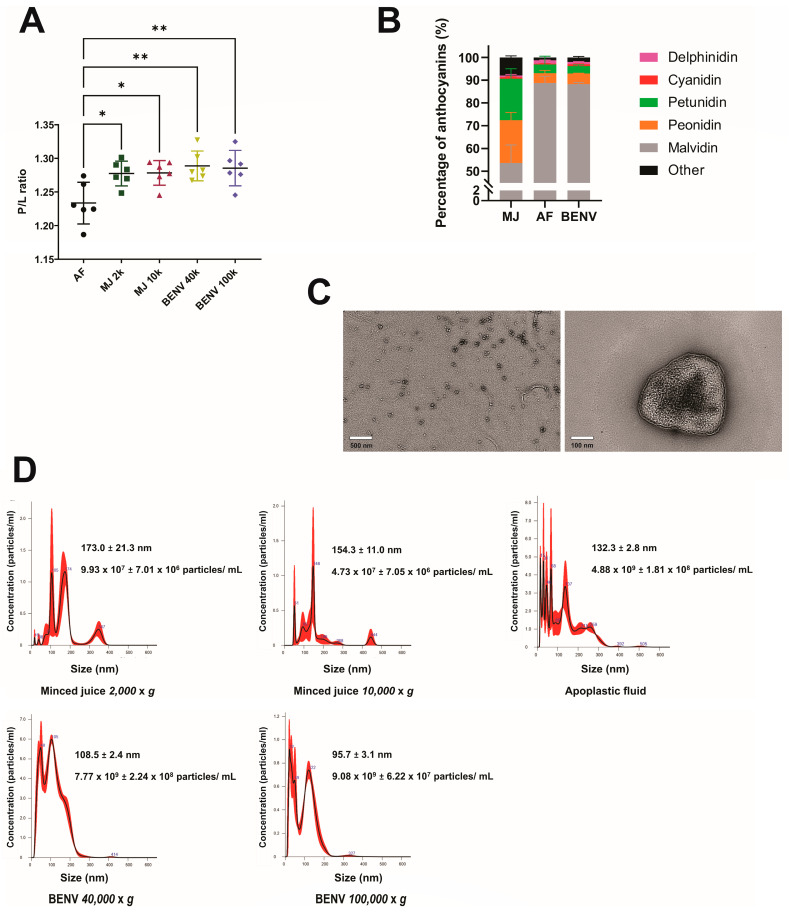
Characterization of blueberry-derived extracellular nanovesicles. (**A**) FTIR spectroscopic protein-to-lipid ratio (P/L ratio) calculated by the relative intensities of amide I to CH_2_/CH_3_ stretching of apoplastic fluid (AF), minced juices (MJ) collected at different centrifugation forces (2000× *g* and 10,000× *g*), and BENVs collected at different centrifugation forces (40,000× *g* and 100,000× *g*). Mean values are represented by horizontal lines, and the standard deviation of the mean is shown as error bars (n = 6). (**B**) Anthocyanin contents in anthocyanin-rich extracts identified by HPLC (n = 3). (**C**) The BENVs collected at 40,000× *g* were stained with phosphotungstic acid to obtain TEM images. (**D**) Particle sizes of anthocyanin-rich extracts collected from different centrifugation forces using NanoSight NS300 (n = 3). *, *p* < 0.05 and **, *p* < 0.01.

For the analysis of BENVs thus prepared, we employed a FTIR technique as it is efficient in identifying biomarkers in different biological species, particularly for extracellular vesicles, based on the “spectroscopic” protein-to-lipid ratio (P/L ratio) [29]. Specifically, it was utilized to distinguish between apoplastic fluid (AF), blueberry minced juice (MJ) collected at 2000× *g* and 10,000× *g* (i.e., MJ 2k and MJ 10k, respectively), and blueberry-derived extracellular vesicles collected at 40,000× *g* and 100,000× *g* (i.e., BENV 40k and BENV 100k, respectively) (Figure S2). To facilitate the analysis, all input samples were adjusted to the same protein concentration prior to FTIR study. As shown in Figure 1A, MJ and BENVs showed higher P/L ratio compared to that in AF, indicating the lower lipid concentrations in these samples. It is most likely that the AF is enriched with the cell wall’s lipids when it flows through the 3D filter [35]. Moreover, the lipid contents in the MJ could be influenced by damaged cells or fragmented cells generated from the grinding or juicing process. Thus, BENVs contained lower lipid concentrations than the intact fruit, resulting in a higher P/L ratio at the same input concentration, indicative of the success of our novel 3D filter-based isolation method. 

Next, the total anthocyanin content in each sample, including AF, MJ 10k, and BENV 40k, was determined by HPLC (Figure S3). Interestingly, AF and BENV were found to contain anthocyanin, which was mainly comprised of malvidin, approximately 88% of the total anthocyanins (Figure 1B). Meanwhile, malvidin accounted for more than half of the total anthocyanins in MJ (53.65%), followed by peonidin (18.73%) and petunidin (18.28%). The presence of delphinidin and cyanidin in all three samples was hardly noticeable. The higher percentage of anthocyanin compounds in BENVs compared to that in AF might result from the sedimentation of the smallest non-EV structures (e.g., exomeres and high-density lipoprotein) upon extended high-speed centrifugation [36]. This hypothesis was supported by NTA data in which an abundance of uncharacterized small particles ranging from 10 nm to 30 nm was detected in BENV samples collected at 40,000× *g* and 100,000× *g* (Figure 1C). Remarkably, the particle numbers of the BENVs isolated from apoplastic fluid at different centrifugal forces were significantly enriched to 7.77 × 10^9^ ± 2.24 × 10^8^ particles/mL and 9.08 × 10^9^ ± 6.22 × 10^7^ particles/mL for BENVs collected with a centrifugal force of 40,000× *g* and 100,000× *g*, respectively (Figure 1C). A small number of BENV particles ranging from 300–450 nm was also counted as EVs, corresponding to a study by Xiao et al. [37]. The 2D and 3D morphology of BENVs were studied by TEM and AFM, respectively (Figure 1D and Figure S4). BENVs exhibited a lipid bilayer typical of extracellular nanovesicles and were surrounded by a network of extravesicular channels. This is consistent with a study performed by Sharma et al., in which the AFM image of saliva EVs displayed a similar channel network [38]. Moreover, the protein content of BENVs was found to be 6174.04 ± 68.58 mg per liter, which is 18-fold higher than those collected from milk EVs and 3000-fold higher than EVs collected from cell culture supernatant (Table S1), indicating a high yield of our isolation method for drug delivery purposes [39,40].

### 3.2. A putative “Universal” Biomarker of Plant-Derived Extracellular Nanovesicles

**Figure 2:**
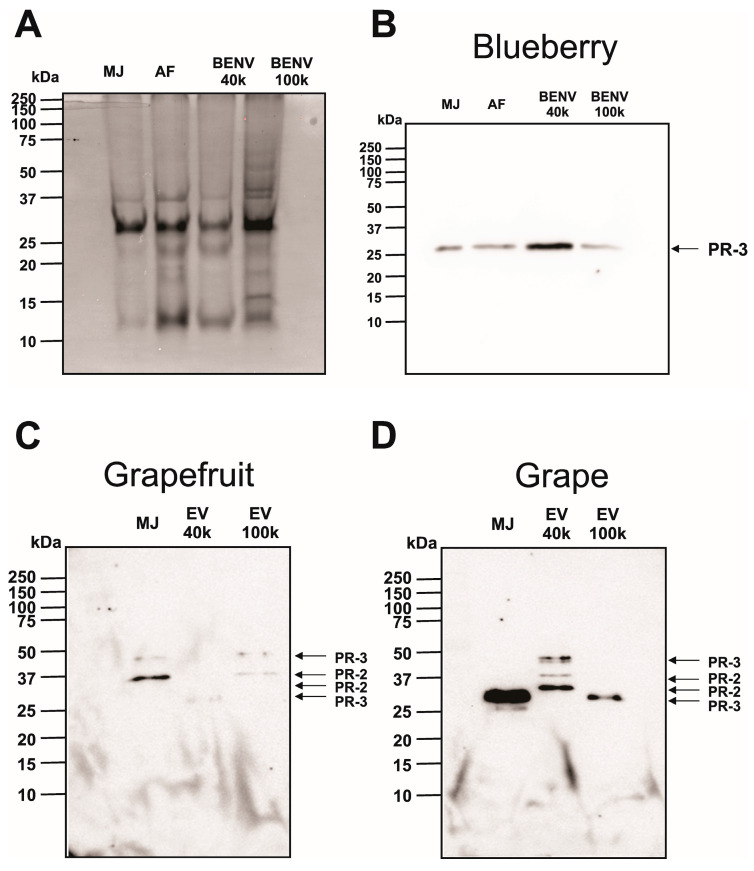
Analysis of proteins in blueberry-derived extracellular nanovesicles. (**A**) Coomassie staining of 12% SDS-PAGE gels of protein extracts (17.3 µg/lane) prepared from anthocyanin-rich extracts. Immunodetection of extracellular vesicles extracted from different succulent fruits such as blueberry (**B**), grapefruit (**C**), and grape (**D**) using class I β-1,3-glucanase (PR-2) and class I chitinase (PR-3) antibodies. The isozyme of PR-2 and PR-3 are identified by black arrows. A total of 16.2 µg protein was loaded into each lane. Data shown are representative of three independent experiments.

To the best of our knowledge, there are only a handful putative plant-derived EV biomarkers described so far, including Helija [41,42], TET8 [43], and AtTET8-GFP [44]. However, there is no commercial antibody available for those putative biomarkers, hindering the progression of plant-derived EV research and development. Therefore, we set out to investigate biomarker(s) for plant-derived EVs in general and for BENVs in particular. To this end, the protein species in various fractions of blueberry EV preparation were initially visualized in Coomassie blue-stained gel (Figure 2A). The nature of the proteins of interest found in AF, MJ, and BENVs were investigated further by mass spectrometry to identify possible protein sequences (Supporting Information, Materials and Methods). Based on the mass spectrometry results, the protein of 28 kDa was identified as acidic endochitinase, which is a defensive protein released upon the invasion of fungal pathogens. Previous studies on exosome-like nanovesicles isolated from ginger [45], shiitake [46], and citrus [47] also reported the presence of a protein with similar molecular weight in the SDS-PAGE analysis, though the identity of such protein remains elusive. As EVs are originally attributed to the interactions between plants and pathogens, we hypothesized that pathogen-related (PR) proteins could be sorted onto the EV surface and thus serve as biomarkers for plant-derived EVs. To test this hypothesis, we proceeded to examine the presence of pathogen-related (PR) proteins such as class I β-1,3-glucanase (PR-2), class I chitinase (PR-3), pathogenesis-related protein 5 (PR-5), and isoflavone reductase (IFR) in different fruit samples (i.e., blueberry, grapefruit, and grape) to identify possible biomarkers. A complete blot of the SDS-PAGE analysis is shown in Figure 2. Among them, class I chitinase (PR-3) was present in all samples and highly accumulated in EV samples, either at the position of 25 kDa or 38 kDa upon Western blot analysis (Figure 2B–D). However, grapefruit EVs extracted at 40,000× *g* displayed a very faint band of PR-3 (Figure 2C), which could be explained by the fact that, although the protein concentration of grapefruit EVs is similar to that in other samples, the juicy nature and the contaminated fragments generated during the squeezing method might affect the protein composition of collected EVs. Moreover, grapefruits were reported as cold-sensitive species that developed chilling injury symptoms under low-temperature storage conditions (i.e., lower than 8–10 °C) [48]. In addition, Porat et al. described low levels of chitinase in flavedo tissue of grapefruit in nontreatment conditions in comparison with stress conditions such as wounding and UV treatment, whereas β-1,3-glucanase (PR-2) expression remained stable with or without stress conditions [49]. This could account for the low chitinase expression in all grapefruit samples. Notably, in all samples, there was an accumulation of PR-3 in EVs extracted at 40,000× *g* in comparison with MJ at different molecular weights because they exist in different isozymes (black arrow). For instance, at 40,000× *g*, blueberry EVs and grape EVs showed a prominent protein at 25 kDa, while grapefruit displayed a major protein at 38 kDa. All EVs isolated at 100,000× *g* contained proteins similar to those of the original MJ and/or AF. Another PR protein that was detected in grape and grapefruit-derived EV samples is class I β-1,3-glucanase (PR-2). With an apparent molecular mass of 30 kDa [50], PR-2 was accumulated in grape-derived EVs isolated at 40,000× *g*, while PR-2 isozyme located at approximately 33 kDa [51,52] was observed as faint bands in both grape-derived EVs extracted at 40,000× *g* and grapefruit-derived EVs extracted at 100,000× *g*. This concords with a previous study in which only trace amounts of PR-2 and PR-3 were detected in intracellular and intercellular sites of healthy plants, and the increased expression of PR proteins frequently relates to pathogen attack [20,51,53]. Hence, pathogen-related proteins, such as PR-2 and PR-3 identified here, could be utilized as promising plant-derived EV biomarkers and be used for tracking biogenesis pathways that drive plant-derived EVs to designed locations.

### 3.3. BENVs as a Nanocarrier for Drug Delivery

**Figure 3:**
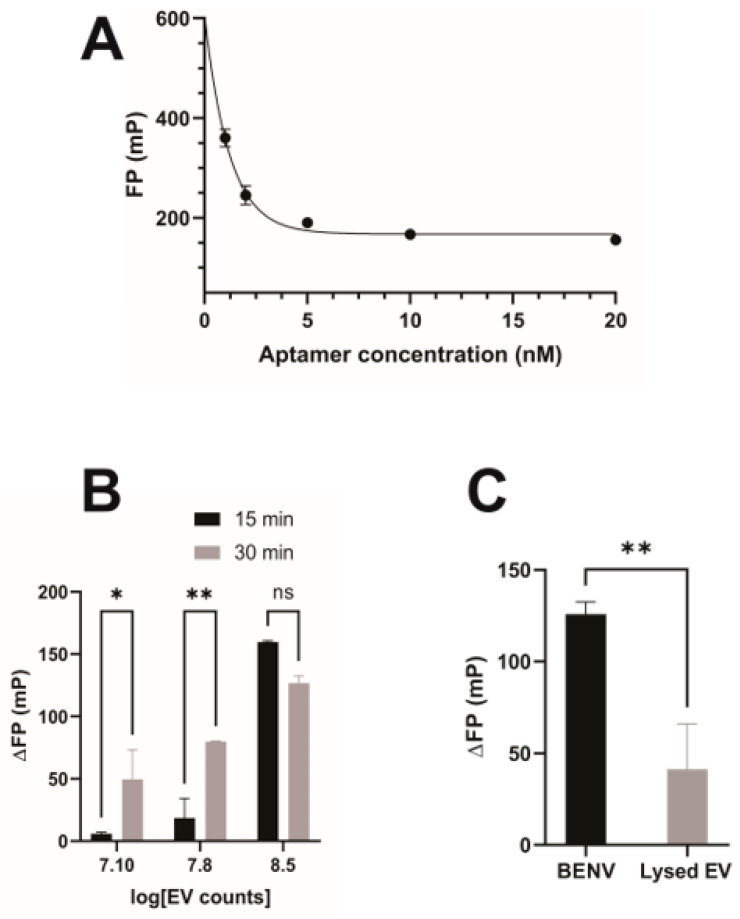
Characterization of conjugation of FAM-labelled EpCAM aptamer with BENV surface using fluorescence polarization. (**A**) Fluorescence polarization of FAM-labelled EpCAM aptamer at different concentrations. (**B**) Effect of incubation time on ΔFP. (**C**) Comparison of the conjugation of FAM-labelled EpCAM aptamer BENV surface to that of BNEV lysed by 0.05% Triton X-100. ns, a statistically non-significant difference. Data shown are means ± S.D., n = 3, *, *p* < 0.05, and **, *p* < 0.01.

Due to the absence of biomarkers, the application of plant-derived EVs in the pharmaceutical industry is hampered by the inability to identify and detect the plant-derived EV itself and demonstrate the binding of plant-derived EVs to target cells or tissues. Herein, we used anti-EpCAM aptamer (SYL3C) as a model EV tracker to investigate the incorporation of modified aptamers to the lipid bilayer membrane of BENVs (Figure S5). The SYL3C aptamer was labelled with 6-FAM (6-Carboxyfluorescein) at the 5′- end to generate a fluorescently tagged version of the aptamer for the BENV trafficking study. The 3′ end of the aptamer was conjugated with TEG-cholesterol, allowing it to insert into the BENV membrane through hydrophobic interactions between the lipid-PEG linker and the phospholipid bilayer [54]. Furthermore, cholesterol-PEG possesses superior characteristics for drug delivery systems (DDS), such as enhanced fluorescence intensity of labeled cells, prevention of the self-assembly of micelles/liposomes, and higher rigidity and stability [54]. Fluorescence polarization (FP) was used to investigate the binding capacity of the anti-EpCAM aptamer and BENVs as the bound and the free form of fluorescently labelled aptamer can easily be analyzed in solution using FP. As shown in Figure 3A, when the aptamer concentration was higher than 5 nM, the FP value was constant (approximately 185 mP), indicating a stable conjugation of the aptamer into BENVs [55]. Encouragingly, ΔFP increased when the BENV concentration increased from 1.2 × 10^7^ particles/mL to 3 × 10^8^ particles/mL (Figure 3B). Moreover, a higher ΔFP was observed after a 30-min incubation compared to that with a 15-min incubation, indicating time-dependent binding, as aptamers had sufficient time to insert into the BENV membrane. At a BENV concentration of 3 × 10^8^ particles/mL, the binding reached its plateau with minimal difference in ΔFP between the two incubation times. To demonstrate that the increase in ΔFP was derived from the decoration of aptamers onto the EV surface and not from the attachment of aptamers to non-vesicle particles in the detection buffer, BENVs were treated with 0.05% Triton X-100 to lyse the vesicles. Indeed, the intact BENVs presented a high ΔFP value because the binding between aptamer and BENV decelerates the rotation speed of the aptamer (Figure 3C). On the contrary, lysed BENVs contained numerous small fragments and exhibited a low ΔFP value, which revealed either no binding or fast diffusion motion of the aptamer-bound fragments, as the molecular mass of lysed BENV fragments treated by detergent have minimal impact on the molecular mass/volume ratio of the aptamer.

**Figure 4:**
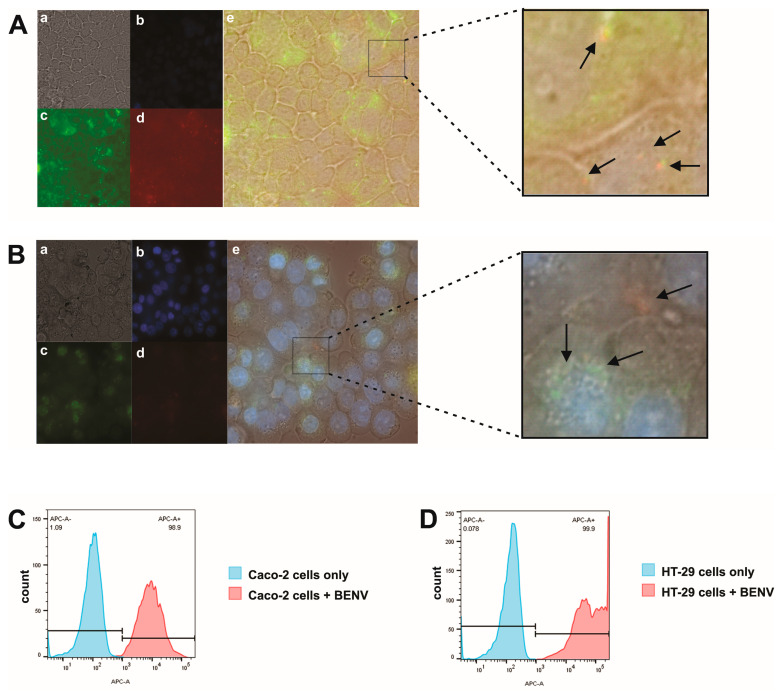
Cellular uptake of dual labelled BENVs in Caco-2 cells and HT-29 cells: (**a**) Bright field, (**b**) Hoechst 33342-stained cells, (**c**) FAM-labelled BENVs uptake by cells, (**d**) DiD-labelled BENV uptake by cells, and (**e**) I merged images. BENVs are labelled with lipophilic carbocyanine membrane dye—DiD (red) and FAM-cholesterol EpCAM aptamer (green). Caco-2 and HT-29 cells were incubated with dual labelled BENVs for 6 h at 37 °C, prior to analysis by Nikon Ti2 microscope (Magnification: 100×) (**A**,**B**) and flow cytometry (**C**,**D**). Data are representative of three independent experiments. Arrows: endocytosed BENVs.

Next, we investigated the cellular uptake of BENVs. To this end, lipophilic carbocyanine membrane dye DiD (red) was incorporated into the BENV membrane and the FAM-anti-EpCAM-Chol aptamer (green) was conjugated onto the BENV surface to produce a dual-fluorescence label. The dual-labelled BENVs were found to be completely internalized into Caco-2 and HT-29 cells (Figure 4A,B) after 6 h of incubation. The colocalization of red (DiD, for BENV’s lipid bilayer membrane) and green (FAM, for EpCAM aptamer) indicates the integrity of BENVs after internalization. Interestingly, BENVs tended to accumulate in the cytoplasm of Caco-2 cells, suggesting that BENVs might be taken up by Caco-2 cells via receptor-mediated internalization [56]. Remarkably, BENVs were found to selectively distribute to the nuclear region of HT-29 cells. Since Caco-2 cells exhibit low cancer phenotypes in comparison with bona fide high grade colorectal adenocarcinoma cells such as HT-29, it would be interesting to explore if BENVs could preferentially target susceptible cells [57]. The cellular uptake results were also confirmed by flow cytometry in which all human cancer cells contained fluorescence-labelled BENVs after 6 h (Figure 4C,D). Additional studies are planned to explore the mechanism of uptake.

**Figure 5:**
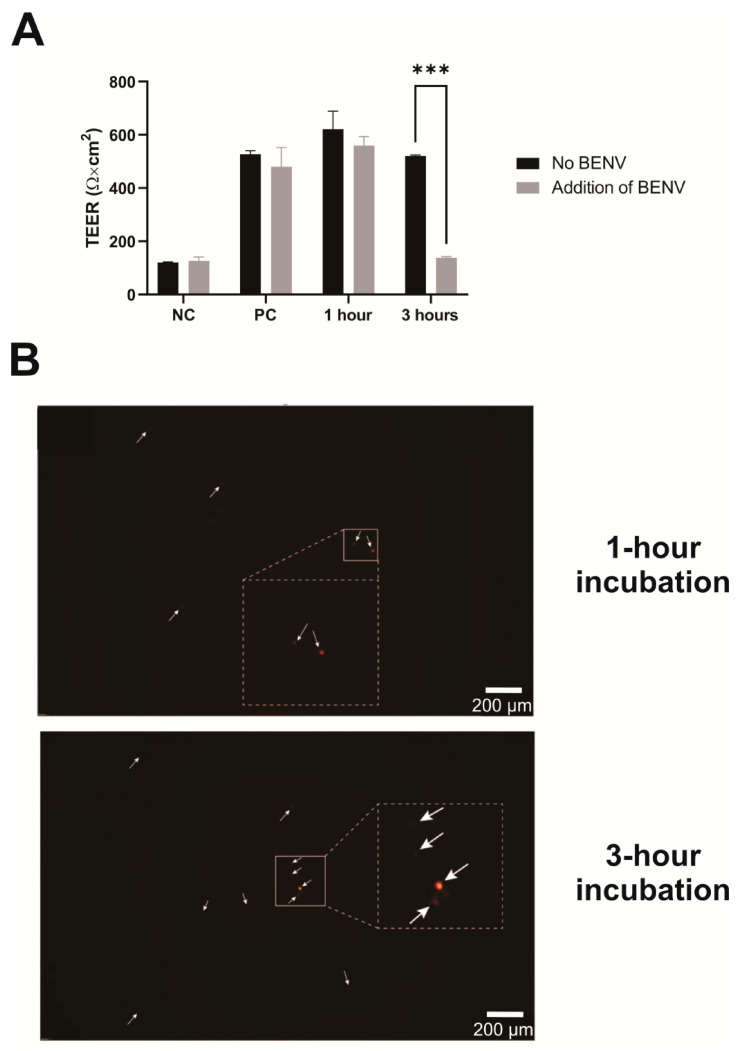
Time-dependent translocation of intact BENVs across Caco-2 cells monolayer. (**A**) Transepithelial electrical resistance (TEER) values of Caco-2 cell monolayers before and after exposure to CUR-loaded BENVs labelled with DiD (red) for 1 h and 3 h. NC, the membrane filter without cells as a negative control. PC, the Caco-2 cell monolayer without CUR-loaded BENV’s treatment as a positive control for TEER. Data are shown as means ± SD (n = 3). ***, *p* < 0.001. (**B**) Visualization of DiD-labelled BENVs collected from the basal compartment after 1-h (up panel) and 3-h (bottom panel) incubation using epifluorescence microscopy. Arrows: DiD-labelled BENVs. Data are representative of three independent experiments.

Generally, the small size of BENVs, i.e., approximately 100 nm, allows them to extravasate and translocate through physical barriers as well as travel through the extracellular matrix [58]. We performed a transport study to examine the capability of BENVs to deliver payload across epithelial cell barriers. Figure 5A presents the TEER values of Caco-2 cell monolayers before and after being exposed to CUR-loaded BENVs labeled with DiD (red) for 1 h and 3 h. TEER values before treatment were found to be 570 ± 80 Ω·cm^2^, which is indicative of a good barrier integrity of the Caco-2 cell monolayer (>260 Ω·cm^2^) [59]. Cell monolayers with TEER values lower than 260 Ω·cm^2^ were discarded. As shown in Figure 5A, there were only minor changes in the TEER value after 1 h of incubation with BENVs, indicating that BENVs could be transported across the epithelial monolayer without compromising the integrity of the epithelial cell barrier. Nevertheless, 3 h of incubation with a high concentration of BENVs (10^8^ particles/mL) resulted in a disruption of tight junction integrity leading to leakage of BENVs. Intact BENVs with a spherical shape were detected in the lower chamber after 1 h of incubation. Additionally, observation revealed the presence of more BENVs in the lower chamber after 3 h of incubation, confirming the opening of tight junctions. The disruption of tight junctions could occur during reversible tight junction opening to facilitate the transcellular permeability, which could be recovered after the removal of BENV solution. Based on the results from the cytotoxicity test, it is unlikely that BENVs caused the irreversible disruption on Caco-2 cells as cell viability remained at high values after 24- and 48-h incubation (Section 3.4). Although the appropriate amount of BENVs per specific area (cm^2^) was yet to be determined in this study, the transport study revealed that BENVs could retain the encapsulated materials after crossing the epithelial barrier without causing membrane disruption for 1 h.

**Figure 6:**
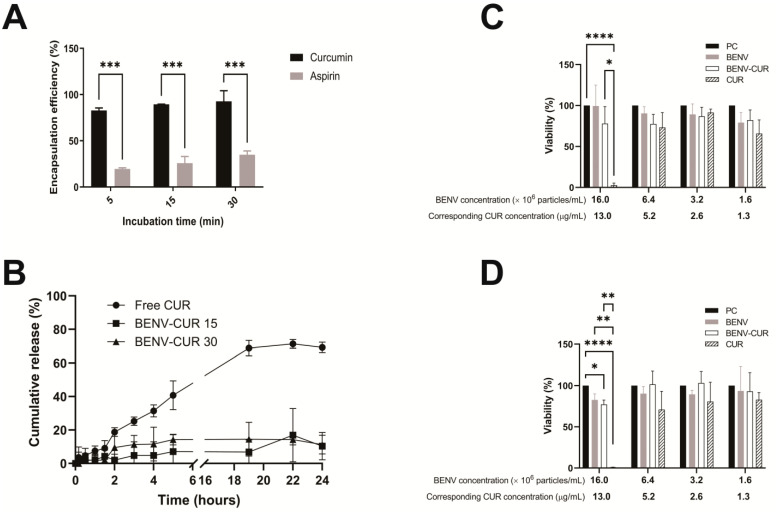
Drug release and safety profile of curcumin-loaded BENVs. (**A**) Encapsulation efficiency of BENVs loaded with curcumin and aspirin over different incubation times. (**B**) Drug release profiles of free curcumin (Free CUR), CUR-loaded BENVs for 15 min (BENV-CUR 15), and CUR-loaded BENVs for 30 min (BENV-CUR 30) in vitro. Samples were incubated in a buffer of pH 1.2 for 2 h before transferring to a buffer of pH 6.8 for 22 h. Cytotoxicity assay (MTT assay) for Caco-2 cells treatment were identified after incubating Caco-2 cells with free curcumin (CUR), BENVs, and curcumin-loaded BENVs (BENV-CUR) for 48 h (**C**) and 72 h (**D**). The concentration of free CUR was equivalated to the CUR concentration encapsulated in BENVs. PC: cells without drug treatment. Data shown as means ± S.D., n = 3. (*, *p* < 0.05; **, *p* < 0.01, ***, *p* < 0.001, ****, *p* < 0.0001).

Next, we evaluated the loading capacity of BENVs. In these studies, aspirin and curcumin, well-known hydrophobic drugs that possess cancer chemopreventive and therapeutic effects, were utilized as a model payload. Different factors (such as carrier condition, shaking power, and incubation time) that influenced drug loading efficiency during incubation were examined to determine the optimal loading conditions for BENVs (Figure S6). Even though the encapsulation efficiency increased over time, however, there was only a moderate increase in drug loading from 5 min incubation to 30 min incubation. As shown in Figure 6A, BENVs possessed a high EE for curcumin (approximately 82.76% after 5 min of incubation), which had a tendency of increasing over time, i.e., from 89.41% at 15 min to 92.68% at 30 min. This was a remarkable result because the current loading efficiency of EVs for curcumin is reported to be 18–24% by conventional incubation methods [60]. However, it is clear that the encapsulation efficiency of curcumin is significantly higher than that of aspirin despite of the comparable input amount, namely 120 µM of curcumin and 100 µM of aspirin per 10^11^ BENV particles. The amount of drug loaded into BENVs is significantly higher than those in recent studies on drug-loaded EVs, in which the amounts of doxorubicin and paclitaxel loaded were approximately 0.03 µM and 0.03 µM per 10^11^ EV particles, respectively [61]. 

Oral administration is the route used for approximately 60% of commercial drug products [62]. The main challenges associated with oral DDSs are the harsh environment of the gastrointestinal tract (GIT) and the residence time required for complete absorption [63]. In fact, the GIT confronts oral controlled release formulations with its unique physiological properties, leading to a fast release rate, drug degradation, or pre-systemic clearance [64]. To demonstrate that BENVs can serve as nanocarrier platforms, we investigated their stability at different pH levels, drug release profiles, and cytotoxicity. As shown in Figure S7, the particle sizes of BENVs were found to remain stable in simulated gastric fluid for 2 h, followed by simulated intestinal fluid for the following 22 h. However, particles were prone to aggregation when incubated in water. The zeta potential in simulated gastric fluid increased from −15.6 mV to −2.05 mV, whereas in the simulated intestinal fluid, the zeta potential was greatly reduced to −52.3 mV (Figure S7). Figure 6B shows the in vitro drug release profile of free curcumin and curcumin loaded BENVs at 24 h. During the first 2 h in simulated gastric fluid, 18.82% of the free curcumin passed through a dialysis membrane and was detected in the dialysate. On the other hand, curcumin was slowly liberated and dialysed from BENV-CUR 15 and BENV-CUR 30, approximately 2.02% and 9.53%, respectively. After being transferred to simulated small intestinal fluid, free CUR was gradually released and reached a plateau after 24 h (approximately 71.37%). However, BENVs released curcumin slowly at approximately 7.1% and 14.3% for BENV-CUR 15 and BENV-CUR 30 after 19 h, respectively. Interestingly, curcumin incubated with BENVs for 30 min exhibited a higher percentage of drug release than that achieved with the shorter incubation time (15 min). This result could be explained by the different curcumin-to-lipid (C/L) molar ratios. Curcumin molecules tend to associate with the glycerol group at low C/L, whereas at higher C/L, they accumulate closer to the headgroup of the lipids in the membrane leaflet [65]. We speculated that the increase in C/L after a 30-min incubation reorients curcumin molecules towards the headgroup of the leaflet, facilitating the liberation of curcumin from BENV. Nevertheless, unlike the properties demonstrated in the releasing profile of free CUR, BENVs were able to retain most of its payload, facilitating sustained release and accumulation of curcumin at the intended sites of targeted drug delivery. As for their potential toxicity, BENV and CUR-loaded BENV were found to be largely nontoxic to cells (Figure 6C,D and Figure S8). However, the viability of CaCo-2 cells and HT-29 cells was reduced with the increase in free curcumin levels. In addition, HT-29 cells displayed more resistance to curcumin than that of Caco-2 cells, evident from the fact that 13 µg/mL curcumin caused 96.4% and 65.8% loss of cell viability in Caco-2 and HT-29 cells, respectively. Although curcumin at the concentrations of 2.5 and 5 μg/mL induces DNA damage to human hepatoma G2 cells both in the mitochondrial and nuclear genomes [66], cancer cells do not die unless they are exposed to 5–50 μM curcumin for several hours [67,68,69]. In our study, HT-29 cells were able to maintain their viability when they were treated with either BENV or CUR-loaded BENV for 48–72 h. This result could be explained by the ability of BENV to hold its payload once encapsulated, with only 14.3% of curcumin released from BENVs over a period of 72 h. A slow leak of a small amount of encapsulated CUR is insufficient to cause cell death in HT-29 cells. In addition, it is possible that the intrinsic contents of BENVs may counteract the effects of curcumin. 

### 3.4. Immunomodulatory Effects of BENVs

**Figure 7:**
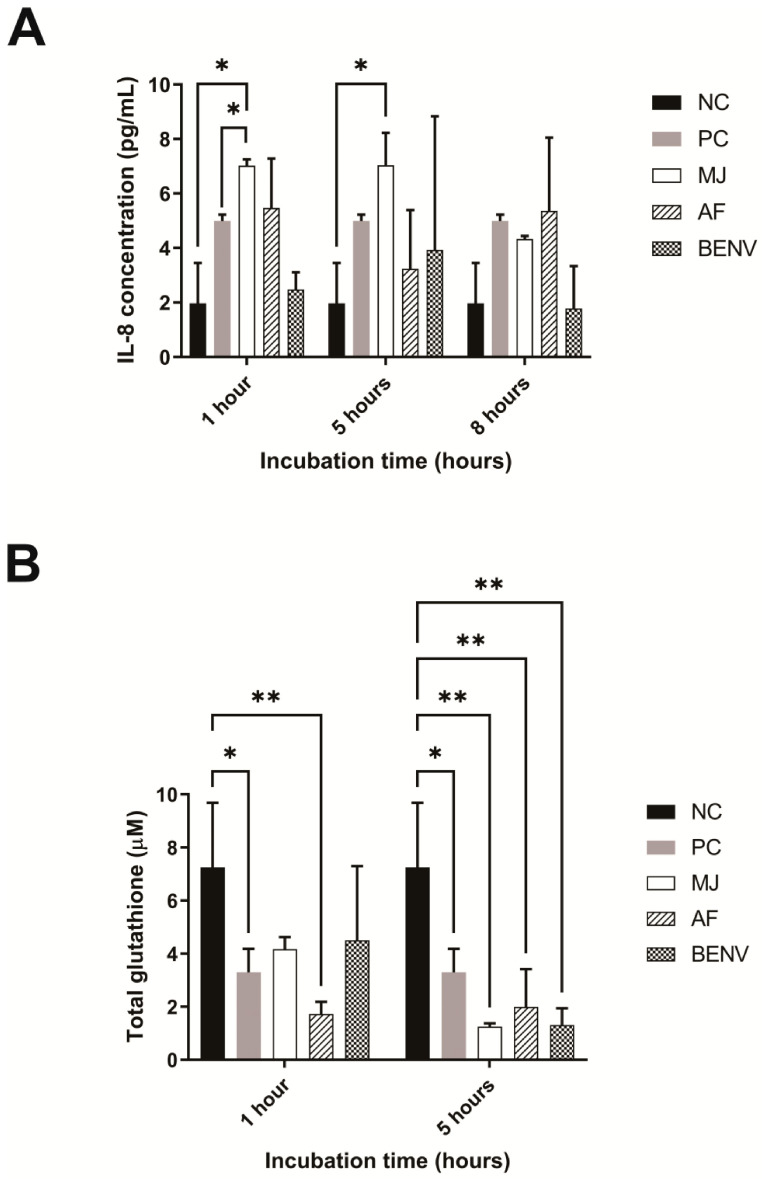
Immunomodulatory effects of anthocyanin-rich extracts. Caco-2 cells were pre-treated with 500 μg/mL extracts as indicated for 1, 5, and 8 h, followed by incubation with 2 mM of H_2_O_2_ for 6 h. The IL-8 released into the supernatant (**A**) or total GSH production in treated Caco-2 cells (**B**) were analyzed. MJ: minced juice; AF: apoplastic fluid, BENV: blueberry-derived EVs; NC: at the end of the 14-h experiment, the supernatant of the control wells containing cell culture medium/PBS without the treatment of fruit extract nor H_2_O_2_ was collected and used for the determination of basal IL-8. For the determination of basal level of GSH, the cells treated with medium/PBS only were collected by the scraping method (Section 2.14); and PC: cells treated with H_2_O_2_ only as a positive control. Data shown as means ± S.D., n = 3. (*, *p* < 0.05 and **, *p* < 0.01).

Oxidative stress upregulates the production of inflammatory cytokines in cells [70]. The gastrointestinal tract (GIT) is vulnerable to exogenous oxidant effects as it serves as the primary digestive system. The GIT is directly affected by various stimuli, such as pollutants, smoking, drugs, xenobiotics, food toxins, heavy metal ions, and intestinal microflora [71]. Although monocytic cells are commonly used to evaluate the immunomodulatory effects, Caco-2 (Cancer Coli-2) was chosen as a cellular model to study the ability of BENVs to regulate inflammation-associated colorectal cancer because of their ability to mimic the intestinal barrier and high sensitivity to H_2_O_2_-induced oxidative stress. Proinflammatory cytokine IL-8 is well-known as an oxidative stress indicator in Caco-2 cells. We hypothesized that the production of IL-8 protein caused by oxidative stress will be suppressed by pre-incubating Caco-2 cells with anthocyanin-rich extracts. Indeed, preincubation of Caco-2 cells with AF and BENVs significantly reduced the IL-8 level caused by H_2_O_2_, whereas MJ only suppressed IL-8 release after 8 h of incubation (Figure 7A). This could be attributed by the malvidin contents in AF and BENVs, which facilitates a faster transportation across the Caco-2 cell monolayer [72]. A short pre-incubation time with MJ (i.e., 1 h or 5 h) had no effect on oxidative stress modulation nor increased IL-8 release (Figure 7A). 

Total glutathione (GSH) is an important indicator of the intrinsic cellular antioxidant response and thus we studied the GSH level to explore the biochemical mechanism underlying the antioxidant effects of BENVs. As shown in Figure 7B, treatment of Caco-2 cells with MJ and BENVs resulted in an elevation of the total GSH after 1 h of preincubation, of approximately 57.5% and 61.6%, respectively, in comparison with that in the control Caco-2 cells. On the other hand, treatment with AF retained a low total GSH level (23.7%) under oxidative stress, which was even lower than that in the positive control cells (54.8%) (Figure 7B). However, pre-treatment of Caco-2 cells by incubating with BENVs or blueberry extracts (i.e., MJ and AF) for 5 h was unable to provoke anti-oxidative activity, evident from the low GSH level. The percentages of recovery of total glutathione of MJ, AF, and BENVs after 5 h of preincubation were 17.1%, 27.4%, and 17.8%, respectively. The low level of GSH in Caco-2 cells after 5 h incubation with anthocyanin-rich extracts compared to that after 1-h incubation could be attributed to the degradation of anthocyanins in culture medium. In addition, although 5-h incubation of anthocyanin-rich extracts such as AF and BENVs could suppress H_2_O_2_-induced oxidative stress, total GSH remained at a low level, indicating that anthocyanin contents in anthocyanin-rich extracts might modulate inflammatory processes at the cytokine level but not at the enzymatic level. It also remains possible that the immunomodulatory effects derive from the combined effects of different constituents that are co-extracted with anthocyanins. 

## 4. Discussion

Currently, the conventional approach for extracting AF from seeds or leaves employs the infiltration method. This method has several limitations, such as bulky installation, tissue shearing, contamination, and unknown impacts of filtration buffers on the obtained EV samples [21]. Herein, we proposed a straightforward method to extract AF and designed a laboratory-scale tool to facilitate the extraction process. We have been successful in isolating BENVs from AF, paving the way to collect EVs with minimal destruction of cells, improving the purity of EVs and facilitating downstream applications. The isolated BENVs exhibited small particle size with remarkably high concentrations of plant-based proteins that could facilitate a plant-based oral drug delivery system [39,40]. 

The discovery of biomarkers for plant-derived EVs constitutes one of the bottlenecks in plant EV research. Our immunoblotting assays revealed the presence of PR proteins in all EV samples (Figure 2), implying that PR proteins could be sorted into EV membranes and fused to the plasma membrane. To test our hypothesis, we investigated the linkage between PR proteins and the EV’s trafficking pathways. In plants, EVs are formed from the TGN (also known as early endosomes), where sorting occurs to release, recycle, and transport the vacuolar membrane [73]. The ESCRT-independent pathway has been implicated in the sorting, revealing that the formation of EVs in the multivesicular body (MVB) lumen continues without the presence of all four ESCRT complexes [74]. Here, we employed an indirect approach to study the EV secretion in which the essential membrane trafficking components participating in MBV formation were investigated [1]. In the conventional pathway, MVBs are fused with the tonoplast or engulfed by the tonoplast, and subsequently release their cargo into the vacuole for degradation. In the unconventional pathway, MVBs are fused with the plasma membrane followed by the releasing of EVs. In this context, the plant EV biomarker proteins might be able to guide EVs either to the vacuole or the pathogenic attack sites. Defense-related proteins, which account for a majority of the proteins of the basal apoplastic proteome, are known to play different roles in plant survival [75]. Among defense-related proteins, pathogen-related proteins are most abundant, accounting for approximately 23–33% of such proteins. However, only 10–15% of the pathogen-related proteins are released into apoplastic fluid under biotic stress [76,77,78]. In susceptible conditions, pathogen-related proteins participate in various defense-related activities, including antifungal [79,80] and cryoprotective functions [81,82]. In healthy plants, pathogen-related proteins are employed to maintain various physiological processes such as material trafficking, flower formation, seed maturation, and ripening [83,84,85,86,87]. It is well-known that PR-3 is involved in the plant defense system alone or in combination with PR-2 [81,88], as well as contributing to growth and developmental processes. Hence, they are present in all organs and plant tissues, including the apoplast and vacuole [88]. Furthermore, class I β-1,3-glucanase and class I chitinase have been shown to be essential determinants for vacuolar sorting machinery [82,89,90,91]. In addition, pathogen-related proteins are transported from the endoplasmic reticulum (ER) to the vacuole thanks to their special peptide structures [89,90]. Family 19 chitinases (i.e., class I, II, and IV chitinases) possess a peculiar C-terminal extension that is crucial for transport to the vacuole [91]. The C-terminal pro-peptides of vacuolar class I chitinase were able to effortlessly enter the sorting machinery and could be eliminated by endo- or exosome-peptidases [91]. On the other hand, class I β-1,3-glucanases contain both an N-terminal hydrophobic signal peptide and an N-glycosylated C-terminal extension at a single site, facilitating the targeting activity of the protein to the vacuole [82,92]. Therefore, the presence of PR-2 and PR-3 proteins in MVB or its pinching EVs could improve vacuolar targeting. Furthermore, pathogen-related proteins participate in the defense system and plant metabolism [75], in which they guide the released EVs to be fused with the plasma membrane and transported through apoplastic fluid to their destinations. Based on the data presented in Figure 2, we proposed that these pathogen-related proteins are sorted onto the EV surface through the ESCRT-independent pathways. Subsequently, the pathogen-related proteins containing EVs either follow the fusion of MVBs to the plasma membrane and are transported to pathogenic sites/neighboring plant cells through the apoplastic pathway or are guided to vacuoles for degradation.

Although EV-based drug delivery possesses various attractive characteristics as cancer therapeutics, two challenges remain in the clinical approach, including low yield and labor-intensive preparation procedures to produce targeted EVs [54]. Until now, there has been only one study that fabricated arrow-tail pRNA-3 WJ and folic acid onto plant-derived EV surfaces for targeted delivery to the tumor site [93]. Herein, we introduce an aptamer design that is straightforward and suitable for large-scale production. Moreover, the characterization of the binding assay was facilitated by the FP technique, which greatly enhances the effectiveness of the procedure. The FP assay showed a significant increase in the FP value in the presence of BENVs, indicating strong binding between the FAM-anti-EpCAM-Chol aptamer and BENVs (Figure 3). To the best of our knowledge, this is the first time that an FP assay has been applied to demonstrate the binding between aptamers and plant-derived EVs, paving the way for investigating the surface engineering of plant-derived EVs, especially when biomarkers of plant-derived EVs remain scant. As the use of plant-derived EVs is still in its infancy, the surface functionalized aptamers would play dual functions: not only targeting cancer cells but also tracking plant-derived EVs along with lipophilic dyes. 

It has been shown that there are at least four different transport mechanisms underlying the uptake and transportation of macromolecules through the epithelial monolayer, including paracellular transport, passive diffusion, vesicle-mediated transcytosis, and carrier-mediated uptake and diffusion [94]. Vashisht et al. visualized the uptake of curcumin-loaded milk-derived EVs in Caco-2 cells using fluorescence microscopy, demonstrating the accumulation of curcumin-loaded milk-derived EVs in the cytosol [95]. Interestingly, we found that BENVs tend to accumulate in the cytoplasm of Caco-2 cells, suggesting that BENVs were taken up by Caco-2 cells via receptor-mediated internalization [56]. Additionally, BENVs are specifically distributed in the nuclear region of HT-29 cells. Therefore, we propose that BENVs might be able to selectively distribute to different subcellular locations depending on the cell type. Previous studies proposed several mechanisms for EV uptake. Tian et al. indicated that actin is involved in EV endocytosis [96]. Actin polymerization participates in various endocytosis pathways including phagocytosis, macropinocytosis, clathrin-mediated endocytosis (CME), clathrin-independent carrier/GPI-anchored protein enriched endosomal compartment (CLIC/GEEC) endocytosis, fast endophilin-mediated endocytosis (FEME) and interleukin-2 receptor (IL2R) endocytosis [96,97]. The latest studies added two endocytic pathways into the system, namely fast endophilin-mediated endocytosis (FEME, a clathrin-independent but dynamin-dependent pathway for rapid ligand-driven endocytosis of specific membrane proteins) and caveolar endocytosis [98]. However, additional experiments are required to demonstrate the uptake mechanisms and endocytosis pathways. The enterocytes account for 90–95% of cell lining in the GIT [99]. The enterocyte barrier-forming Caco-2 cells could facilitate the investigation of BENV absorption through the intestinal wall, and subsequently evaluate the capability of BENVs to enter the systemic circulation and travel to the targeted cancer site. Our results showed that CUR-loaded BENVs could pass through the epithelial monolayer after 1 h without causing disruption to the membrane, demonstrating the potential of BENVs as a promising next generation oral drug delivery system. The potential application of BENVs as nanocarriers to deliver therapeutic substances was further evaluated based on their stability, encapsulation efficiency, and cytotoxicity. One of the prerequisites for BENVs to serve as a nanocarriers for oral DDS is the ability to withstand the harsh environment of orally administered drugs in the GIT. As shown in Figure S7, the isolated BENVs were highly stable in the simulated gastrointestinal tract and displayed a zeta potential value similar to those of previous studies [24,100]. Our BENVs are likely to maintain their particle size despite the change in surface charge, as indicated through experiments performed in simulated gastric solution, simulated intestinal solution, and PBS solution (Figure S7). 

The BENVs exhibited high encapsulation efficiency when the conventional incubation method was used, with values of approximately 36.79% and 82.76% for aspirin and curcumin, respectively. In contrast, recent research has reported that the highest encapsulation efficiency for drug-loaded EVs is 18%, depending on specific drugs as well as the sources of EVs [30,60,61]. Notably, 10^11^ BENV particles can load up to 120 µM of curcumin and 100 µM of aspirin, considerably higher than that achieved with other EV-based drug carriers. For instance, Haney et al. reported that only 0.027 µM of paclitaxel and 0.025 µM of doxorubicin were loaded into 10^11^ macrophage-derived EVs [61]. Intriguingly, curcumin-loaded BENVs had relatively higher encapsulation efficiency than that of currently used curcumin-loaded liposomes, with only 5 min of incubation [101,102]. When inserted into the lipid bilayer, curcumin stays in the lipid tail region, also called the glycerol region, which is near the interface of the lipid head and lipid tail, [103]. Ileri Ercan et al. investigated the distribution of curcumin at different C/L ratios [65]. Curcumin is normally located within the glycerol group at lower C/L; however, an increase in C/L causes the relocation of curcumin towards the headgroup of the lipids [65]. Furthermore, the adjustment of the density distribution in the presence of a higher concentration of CUR likely initiates an energy barrier, which facilitates the penetration of molecules through the bilayers [65]. A previous study showed that 95% of curcumin remained intact in the CUR-DMPC liposome complex after 2 days at pH 6, implying that curcumin is stable in the lipid bilayer [104]. Nevertheless, the loading capacity and encapsulation efficiency are also influenced by the lipophilic characteristics of the drug and concentration gradient [105,106], which require further experiments to optimize. Previous studies showed that the maximum solubility of aspirin in saturated lipid bilayers was 50 mol% aspirin, in which each lipid molecule “hosts” one aspirin molecule to form a non-physiological 2D crystal-like state. Hence, in this study we started with a low dose of aspirin which was 100 µM of aspirin per 10^11^ BENV particles to preserve the lipid bilayer membrane. On the other hand, the mechanism underlying the interaction between curcumin and the BENV lipid bilayer membrane is likely to be similar to that of cholesterol, in which a lower curcumin concentration (<1%) significantly remodels the overall order of the membrane, while a higher curcumin concentration (>1%) induces a decline in the ordering of the glycerol region, followed by the formation of acyl chains [104].

We observed that 7.1% and 14.3% of encapsulated curcumin was released in the pH 6.8 buffer after 19 h for BENV-CUR 15 and BENV-CUR 30, respectively (Figure 6B). A longer incubation time apparently promoted a higher amount of curcumin release, possibly because the high concentration of curcumin caused high disorder in the lipid bilayer over time. Additionally, the increase in curcumin concentration also improved its mobility within the bilayer and created an energy barrier, allowing curcumin molecules to be exposed to more water molecules and thus increasing solvation. High loading capacity, in combination with a prolonged release profile, allows BENVs to become an ideal edible plant-based carrier that reduces dosing cycles and minimizes cytotoxicity [107]. Our MTT results suggest that the encapsulation of curcumin considerably shielded BENVs from the cytotoxicity of free curcumin in both Caco-2 and HT-29 cells (Figure 6C,D and Figure S8). 

Anthocyanin-rich phenolic compounds have been reported as immunomodulatory agents in the human colon adenocarcinoma cell line Caco-2 [34]. In this study, we investigated the effects of BENVs on H_2_O_2_-induced oxidative stress, demonstrating its capability to modulate the immune system. Our results revealed that BENVs suppressed more than 94% of IL-8 release, which is associated with the restoration of cell viability after 6 h of treatment. Long-term exposure to ROS could trigger chronic inflammation and cancerous features in Caco-2 cells. Hence, the suppression of IL-8 by BENVs allows Caco-2 cells to recover with minimal aggressive tumor phenotypes. In previous studies, long-term preincubation was deemed to likely degrade phenolic compounds or convert phenolic-rich compounds into less effective metabolites [34]. However, our results showed that BENVs might overcome the instability of phenolic compounds in cell culture media, resulting in higher cell viability and improved IL-8 suppression (Figure 7). Previous studies showed the intake of approximately 50 to 150 g of fresh blueberries, could contribute to the prevention of type 2 diabetes, neurological decline, and cardiovascular disease [108,109]. In our study, 500 µg/mL BENV proteins extracted from approximately 50 g of fresh blueberry were adequate for the inhibition of IL-8 overproduction, implying that the BENVs could preserve the bioactive properties of blueberries. A previous study showed that the amount of anthocyanin extracted from blueberry transported through a Caco-2 cell monolayer was minimal, approximately 3–4% for averaged transport efficiency [72]. Among them, delphinidin glucoside (Dp-glc) had the lowest transportation/absorption efficiency (<1%), whereas malvidin glucoside (Mv-glc) had the highest. This might explain the greater immunomodulatory effects of AF and BENVs in comparison with MJ, as the malvidin groups were significantly elevated in the obtained AF and BENV, resulting in better transportation and absorption. Although MJ contained a very high amount of peonidin, which would help it absorb through the Caco-2 layer, the anthocyanins extracted from blueberry were drastically degraded in cell culture media (approximately 60% retained in the first hour) [72]. This finding implies that BENVs shelter anthocyanin compounds from degradation, providing long-term proinflammatory effects that none of the current anthocyanin-rich extracts can achieve.

## 5. Conclusions

In this study, we designed an in-tube 3D filter-based approach to extract apoplastic fluid from succulent fruits, using blueberry as a model of edible fruits. This extraction approach is simple, straightforward, and requires no specialized technical skills. Our results demonstrated that apoplastic fluid is successfully extracted from blueberry and the obtained blueberry-derived extracellular nanovesicles contained a significantly higher amount of total proteins in comparison with that in current extracellular nanovesicles extracted from milk and supernatant of mammalian cell culture. Furthermore, plant-based proteins have been shown to assist drug delivery on multiple fronts, enabling future development of BENVs as a novel edible nanocarrier. Additionally, this method preserves the purity of the plant sources and eliminates the interference from extraction buffers or detergents, thus facilitating downstream analysis and applications. Notably, we discovered that pathogen-related proteins (i.e., class I β-1,3-glucanase and class I chitinase) are fused with plant-derived EVs in the transport process, suggesting that these proteins could be used as a potential general biomarker for plant-derived EVs. We show that BENVs possess attractive features of a nanocarrier for drug delivery system, such as incredible stability, low toxicity, low immunogenic effect, high immunomodulatory effect, high cellular uptake, and ability to be transported through the intestinal epithelial barrier. Interestingly, BENVs are able to prolong treatment efficacy by sheltering anthocyanin compounds from degradation caused by culture medium. This characteristic surpasses current anthocyanin-rich extracts. Future optimization of BENVs is likely to make this edible plant EV a multifunctional nanoplatform for targeted drug delivery in immunomodulatory therapy.

## Data Availability

Data are available upon request.

## Supplementary Materials

The following supporting information can be downloaded at: https://www.mdpi.com/article/10.3390/pharmaceutics15082115/s1, Figure S1: An illustration of apoplastic fluid extraction method using homemade in-tube filter to prevent disruption of cell membrane. Figure S2: (A) FTIR spectra of apoplastic fluid (AF), minced juices (MJ) collected at 2000× *g* (MJ 2k) and 10,000× *g* (MJ 10k), and BENVs collected at 40,000× *g* (BENV 40k) and 100,000× *g* (BENV 100k). (B,C) Illustration of selected wavenumber regions for spectral evaluation based on spectroscopic protein-to-lipid ratio (P/L) protocol of Mihaly et al. [29]: (B) Amide group at wavenumber region (1770–1470 cm^−1^) deconvoluted by curve fitting with Lorentz-function (band denoted by dotted lines), (C) C-H stretching region (3040–2700 cm^−1^) indicative for lipid components. The FTIR areas represented amide group and lipid group were calculated by a formula followed Mihaly et al. Figure S3: FTIR profiles of functional groups in 15 possible anthocyanin compounds in the study. Figure S4: Topography of BENVs. Figure S5: Schematic of cancer cell targeting with BENV functionalized with EpCAM aptamer (SYL3C). Figure S6: The effect of experimental conditions on the encapsulation efficiency of BENVs with poorly water-soluble aspirin. Figure S7: The in vitro stability of BENVs under physiological mimetic conditions. Figure S8: Cytotoxicity of BENV and curcumin-loaded BENV in HT-29 cells. Figure S9: Effect of H_2_O_2_ concentration on IL-8 protein secretion in Caco-2 cells. Table S1: Comparison of the protein concentrations in EV samples isolated from different sources. References [29,39,110,111,112,113] are cited in the supplementary materials.
