# Supplementary material for: Development of Blueberry-Derived Extracellular Nanovesicles for Immunomodulatory Therapy
Source: Pharmaceutics. 2023 Aug 10;15(8):2115. doi: 10.3390/pharmaceutics15082115 (PMC10458573; doi:10.3390/pharmaceutics15082115)
Supplement: Supplementary file 1 [file pharmaceutics-15-02115-s001.zip › pharmaceutics-2464390-supplementary.pdf]

Supporting Information

Development of Blueberry-Derived Extracellular Nanovesicles for Immunomodulatory Therapyy

Tuong Ngoc-Gia Nguyen [1], Cuong Viet Pham [1], Rocky Chowdhury [1], Shweta Patel [1], Satendra Kumar Jaysawal [1], Yingchun Hou [2], Huo Xu [3], Lee Jia [3], Andrew Duan [4], Phuong Ha-Lien Tran [1,*] and Wei Duan [1,*]

1. School of Medicine, Faculty of Health Deakin University, Geelong Waurn Ponds Campus, Geelong, VIC 3216, Australia

2. Laboratory of Tumor Molecular and Cellular Biology, College of Life Sciences, Shaanxi Normal Univer-sity, 620 West Chang'an Avenue, Xi'an 710119, China
3. College of Materials and Chemical Engineering, Minjiang University, Fuzhou 350108, China
4. School of Medicine, Faculty of Medicine, Nursing and Health Sciences, Monash University, Clayton, VIC 3800, Australia

* Correspondence: phuong.tran1@deakin.edu.au (P.H.-L.T.); wei.duan@deakin.edu.au (W.D.)

Supporting Information

A. Materials and methods

1. Sample preparation for liquid chromatography – electrospray ionization tandem mass spectrometry

Gel slices collected from Coomassie Blue-stained gels were destained by incubating them in 50 mM NH_4HCO_3 for 5 minutes, following by sonication with 50 mM NH_4HCO_3 in 30% acetonitrile for 15 minutes. Destained solution was eliminated from gel bands by a pulse centrifugation. Next, acetonitrile was added into each tube and incubated for 15 minutes before removed by a pulse centrifugation. Then the gel bands were further dried down by a vacuum centrifugation for 10 minutes.

In-gel reduction was performed by adding 10 mM DTT in 100 mM NH_4HCO_3 to the gel pieces and incubating the samples for 45 minutes at 56 °C. All liquid was removed from the gel by a pulse centrifugation.

In-gel alkylation was performed by adding 55 mM iodoacetamide (IAA) in 100 mM NH_4HCO_3 to the gel pieces and incubating the samples for 30 minutes in darkness at the ambient temperature. All liquid was removed from the gel pieces by pulse centrifugation.

Gel pieces underwent subsequent incubations in 5 mM NH_4HCO_3 for 10 minutes and in acetonitrile for 15 minutes with all liquid removed by a pulse centrifugation between each incubation. The gel pieces were then dried down by a vacuum centrifugation for 10 minutes and were subsequently treated with trypsin (100 ng/µL) and incubated overnight at 37 °C. Peptides were extracted by subsequent 15-min incubations in 50% acetonitrile /1% formic acid and 100% acetonitrile. All peptide-containing liquid was removed from the gel pieces by a pulse centrifugation and dried down to approximately 1 µL by a vacuum centrifugation. Dried-down peptides were reconstituted in 3% acetonitrile /0.1% formic acid for LC-MS/MS analysis.

The original data for proteomic identification of PR-2 and PR-3 are included in the two Excel files named as "Proteomic identification of PR-2" and "Proteomic identification of PR-3" as part of the Supporting Information.

2. Liquid chromatography – electrospray ionization tandem mass spectrometry

Nano-LC−ESI-MS/MS was performed on an Ultimate 3000 RSLC system coupled to a LTQ Orbitrap XL ETD mass spectrometer. Approximately 1.5 µg of each peptide sample was pre-concentrated onto a C18 trapping column (Acclaim PepMap100 C18 75 µm × 20 mm) at a

flow rate of 5 μL/ min in 2% (v/v) acetonitrile 0.1% (v/v) formic acid for 10 minutes. Peptide separation was performed using a 75 μm ID C18 column (Acclaim PepMap100 C18 75 μm × 15 cm, Thermo-Fisher Scientific) at a flow rate of 0.3 μL/min using a linear gradient from 5 to 45% B (A: 5% (v/v) acetonitrile 0.1% (v/v) formic acid, B: 80% (v/v) acetonitrile 0.1% (v/v) formic acid) over 38 minutes, followed by a 2-min wash with 90% B, and an 15-min equilibration with 5%. MS scans were acquired in the mass range of 300 to 2000 m/z at a resolution of 60 000 in FT mode. The 10 most intense precursor ions selected for isolation and were subjected to CID fragmentation using a dynamic exclusion of 5 seconds. Dynamic exclusion criteria entailed a minimum relative signal intensity of 1000, and ≥2+ charge state. An isolation width of 3.0 m/z was used with a normalized collision energy of 35.

3. Data analysis

Raw MS/MS data was converted to MGF file format using Proteome Daemon (v1.3, Thermo Scientific). Acquired data for samples 1 to 5 was searched against all Viridiplantae entries, and samples 6 and 7 were searched against all Homo sapiens entries present in the Swiss-Prot database in Mascot (v2.3.01, Matrixscience). Search parameters were specified as follows: tryptic peptides with a maximum of 2 missed cleavages, peptide mass tolerance of 20 ppm, fragment mass tolerance of 0.8 Da, cysteine carbamidomethylation set as a fixed modification, and methionine oxidation, protein N-terminus acetylation, and deamidation of glutamine and asparagine set as variable modifications.

B. Results

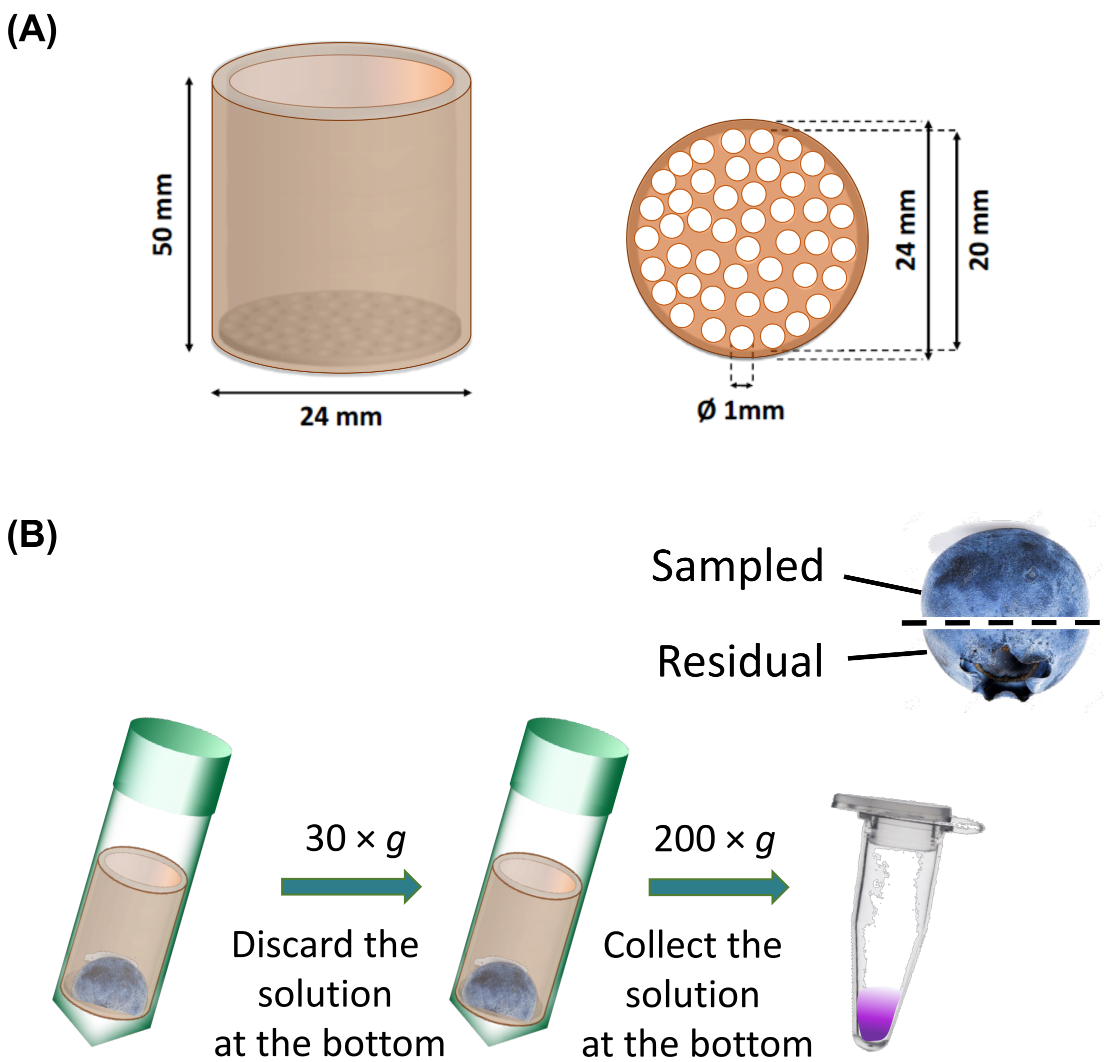

Figure S1: **An illustration of apoplastic fluid extraction method using homemade in-tube filter to prevent disruption of cell membrane.** (A) Three-dimension of inner filter tube made by 3D printing process using PLA filaments. (B) Serial low-speed centrifugations used to prepare the apoplastic fluid.

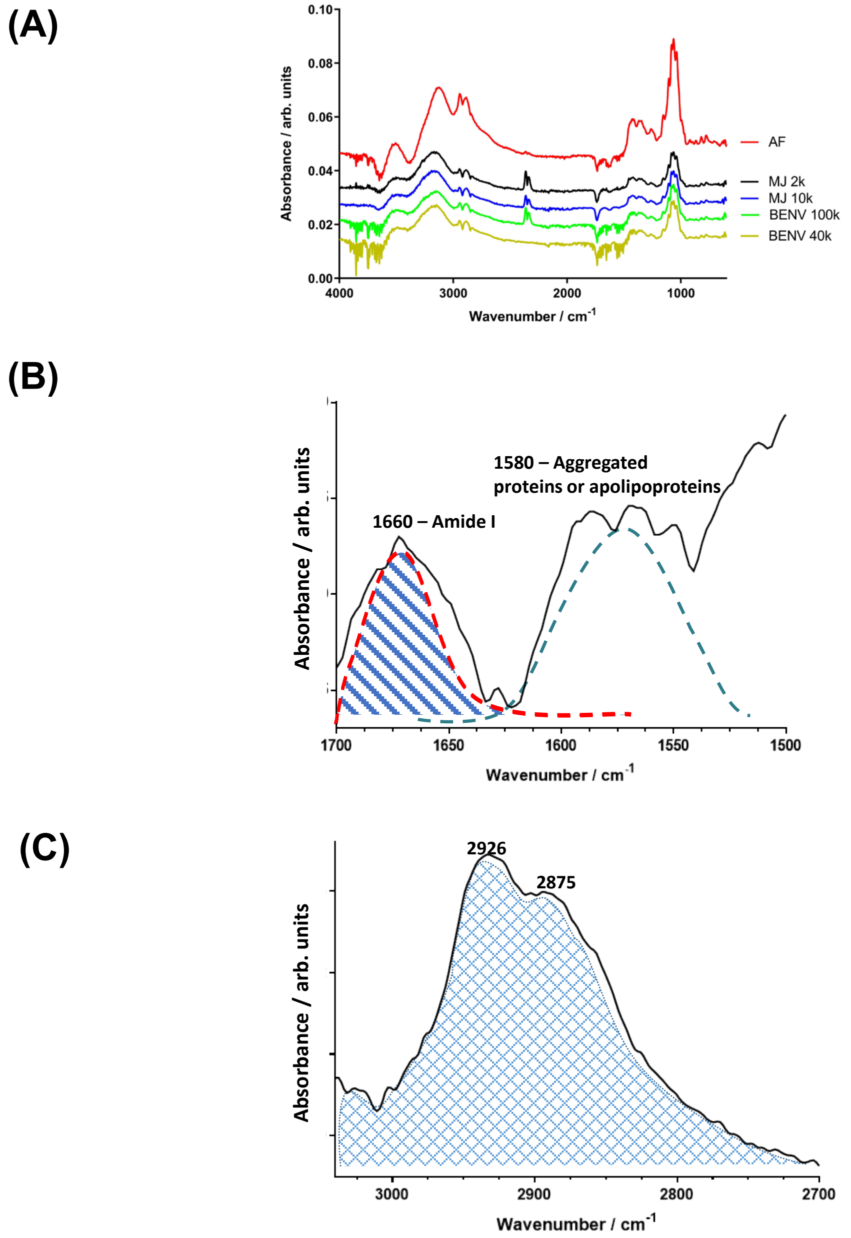

Figure S2: (A) FTIR spectra of apoplastic fluid (AF), minced juices (MJ) collected at 2,000 × *g* (MJ 2k) and 10,000 × *g* (MJ 10k), and BENVs collected at 40,000 × *g* (BENV 40k) and 100,000 × *g* (BENV 100k). **(B, C)** Illustration of selected wavenumber regions for spectral evaluation based on spectroscopic protein-to-lipid ratio (P/L) protocol of Mihaly et al. [1]: **(B)** Amide group at wavenumber region (1770–1470 cm^{-1}) deconvoluted by curve fitting with Lorentz-function (band denoted by dotted lines), **(C)** C-H stretching region (3040–2700 cm^{-1}) indicative for lipid components. The FTIR areas represented amide group and lipid group were calculated by a formula followed Mihaly et al.

Supporting Information

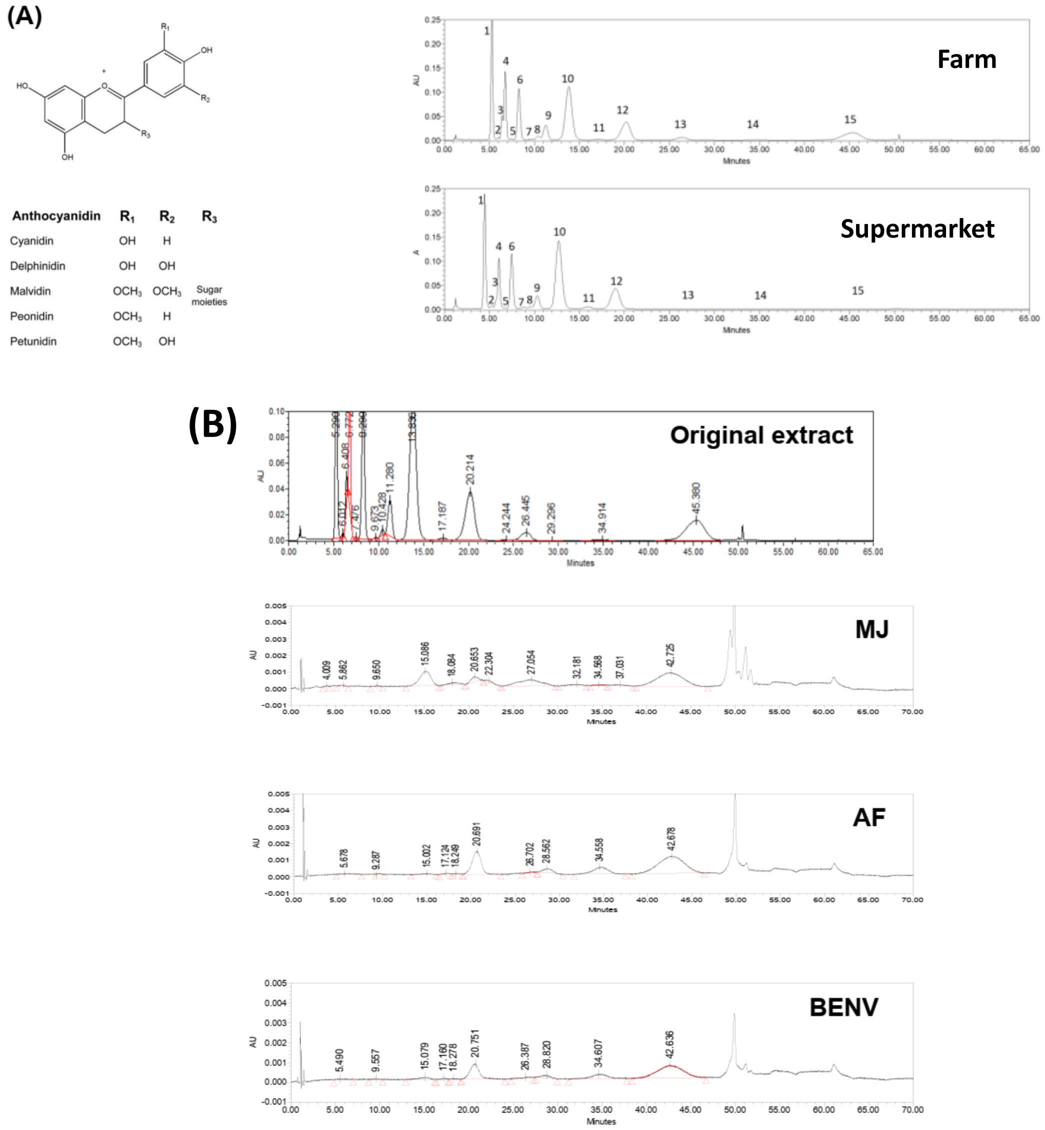

Figure S3: FTIR profiles of functional groups in 15 possible anthocyanin compounds in the study. (**A**) HPLC-PAD chromatograms at 520 nm of the whole fruit extract of blueberry collected from a farm and supermarkets. Peak identification: (1) Delphinidin 3-galactoside, (2) Delphinidin 3- glucoside, (3) Cyanidin 3-galactoside, (4) Delphinidin 3-arabinoside, (5) Cyanidin 3- glucoside, (6) Petunidin 3-galactoside, (7) Cyanidin 3-arabinoside, (8) Petunidin 3- glucoside, (9) Peonidin 3-galactoside, (10) Petunidin 3-arabinoside, (11) Peonidin 3-glucoside, (12) Malvidin 3-galactoside, (13) Peonidin 3-arabinoside, (14) Malvidin 3-glucoside, (15) Malvidin 3-arabinoside. **(B)** HPLC profile of anthocyanins extracts containing 1000 µg/mL protein using the Latti method [2], including blueberry whole fruit, minced juice (MJ), apoplastic fluid (AF), and blueberry-derived extracellular nanovesicles (BENVs). Only peaks that match the anthocyanin peaks of the whole fruit extracted from the Latti method were analyzed. Data are representative of three independent experiments.

Supporting Information

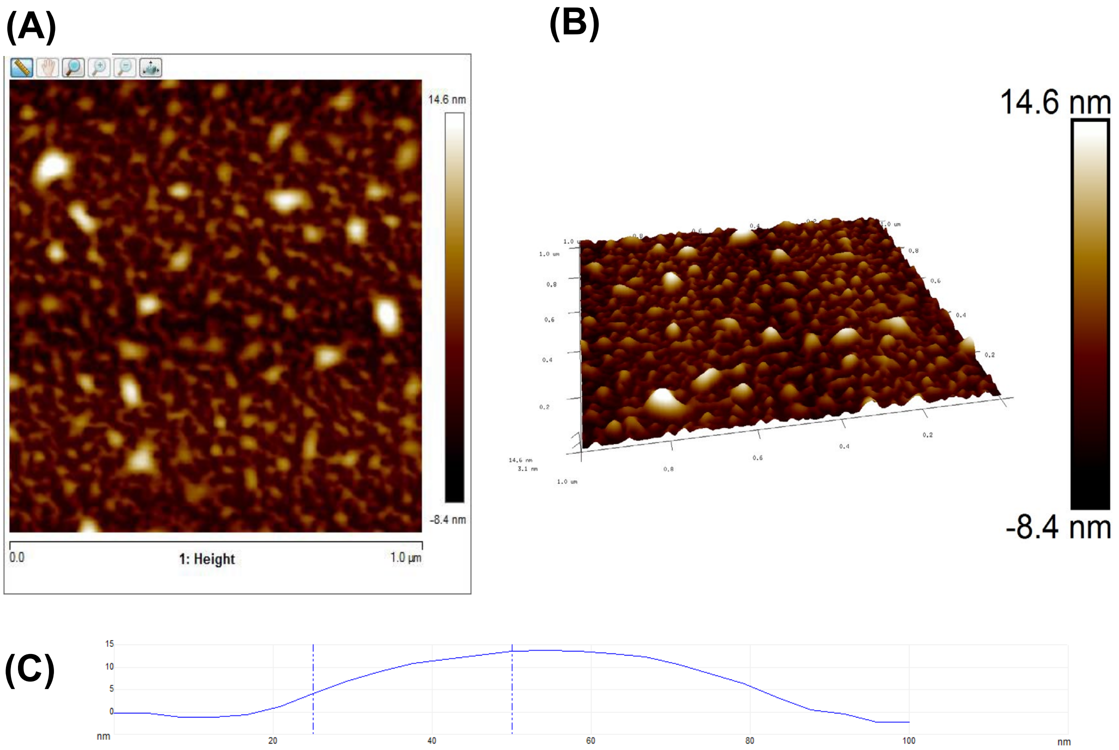

Figure S4: Topography of BENVs. BENVs collected at 40,000 × *g* were diluted 1:50 with deionized water, absorbed on a silicon wafer and analysed using AFM in the range of 30 - 150 nm to generate 2- dimensional (2D) images (**A**) and 3D images (**B**). Size of BENVs was measured by AFM software (**C**). Data shown are representative of three independent experiments.

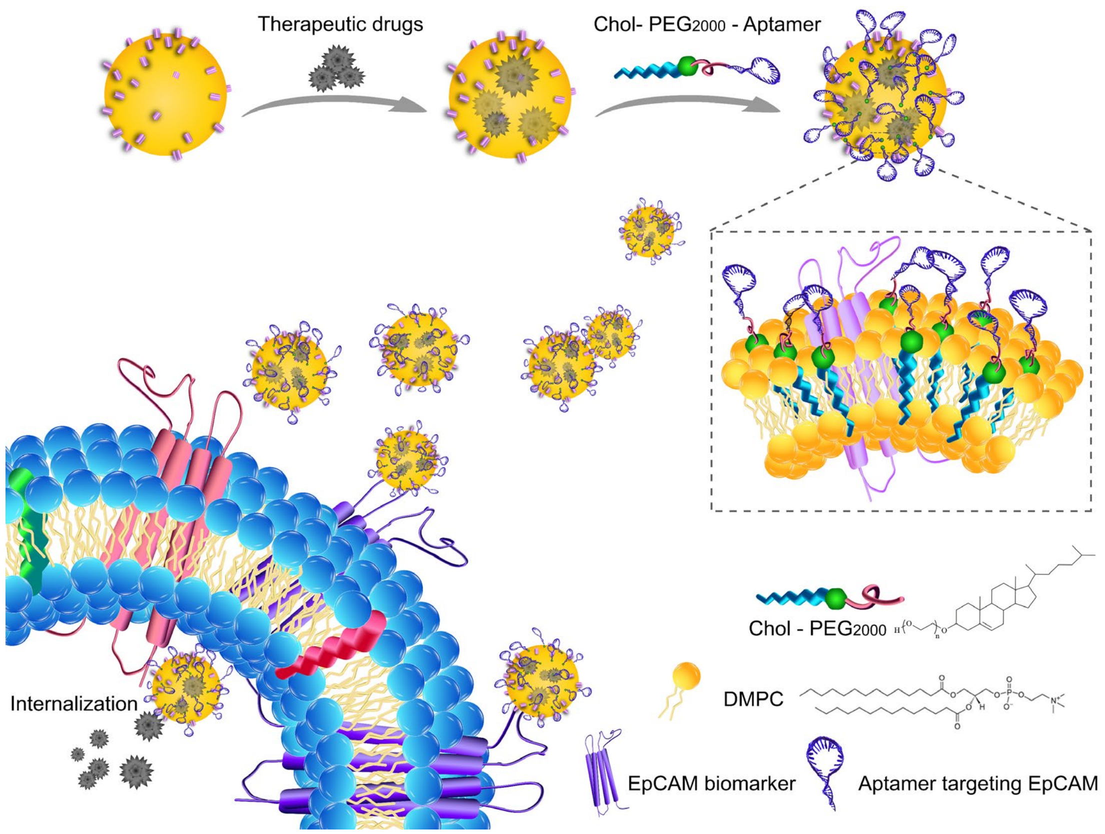

Figure S5: Schematic of cancer cell targeting with BENV functionalized with EpCAM aptamer (SYL3C). The FAM-anti-EpCAM aptamer-is composed of
5′-6-FAM-C*A*C*T*A*C*A*G*A*G*G*T*T*G*C*G*T*C*T*G*T*C*C*C*A*C*G*T*T*G*T*C*A*T*G*G*G*G*G*G*T*T*G*G*C*C*T*G-TEG-Cholesterol - 3′.
The phosphodiester bond of aptamer was replaced by phosphorothioate backbone, providing the protective effects that prevents the aptamer sequence from degradation caused by DNase in plasma. The 3'-end of the aptamer was modified with TEG–Cholesterol (TEG–Chol) to embed aptamer onto BENV's surface through hydrophobic interactions between the lipid-TEG linker and the phospholipid bilayer of BENVs. 6-FAM (6-Carboxyfluorescein) was attached in the 5'- end of the aptamer as a fluorescent reporter. *, the phosphorothioate backbone in the RNA aptamer.

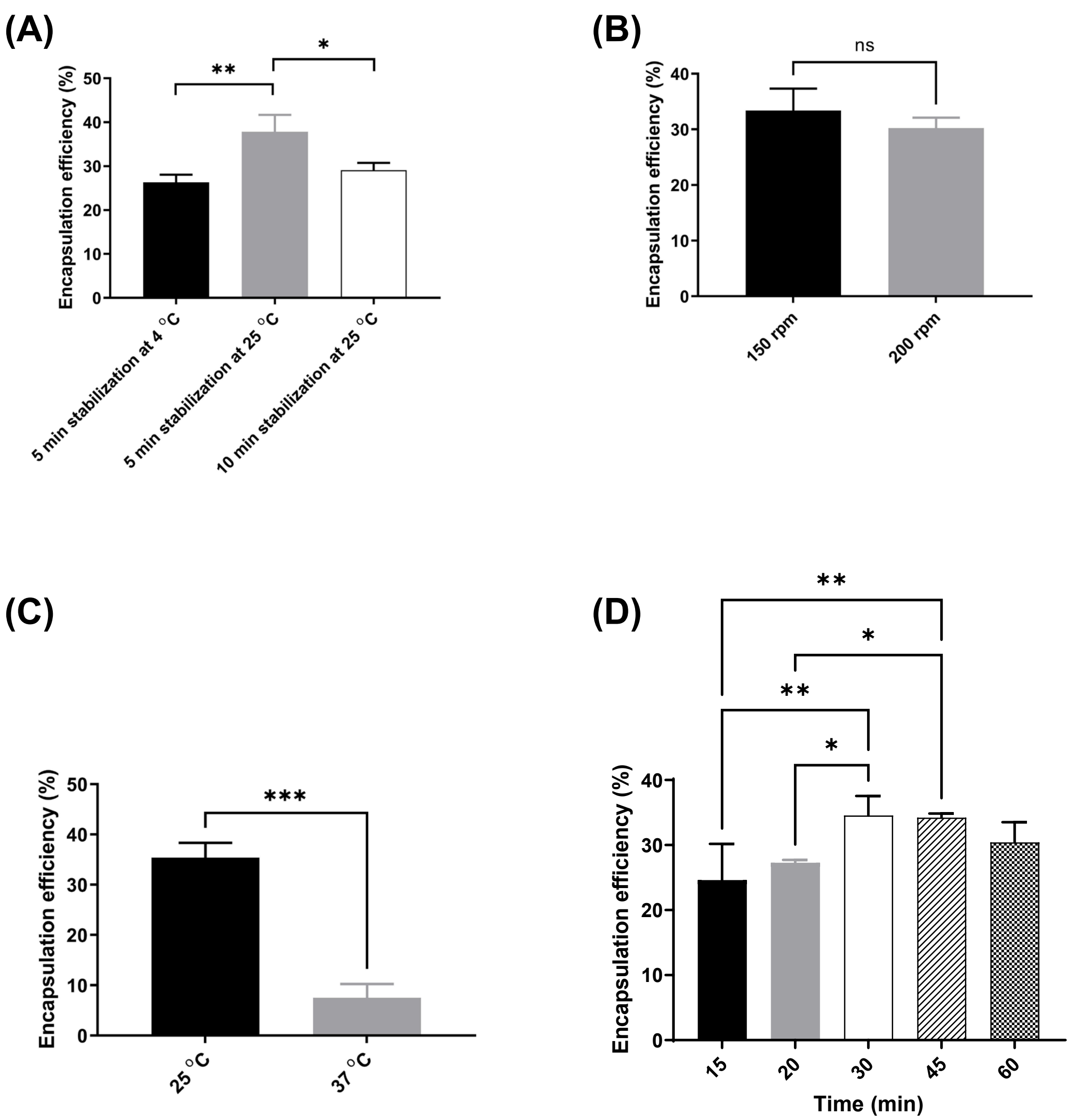

Figure S6: The effect of experimental conditions on the encapsulation efficiency of BENVs with poorly water-soluble aspirin. (**A**) The effect of payload stabilization before loading, (**B**) The effect of incubation temperature during drug loading; (**C**) The effect of shaking power during drug loading; and (**D**) the effect of incubation time during drug loading. Data shown are means ± S.D., n=3. (**p* < 0.05; ***p* < 0.01; ****p* < 0.001).

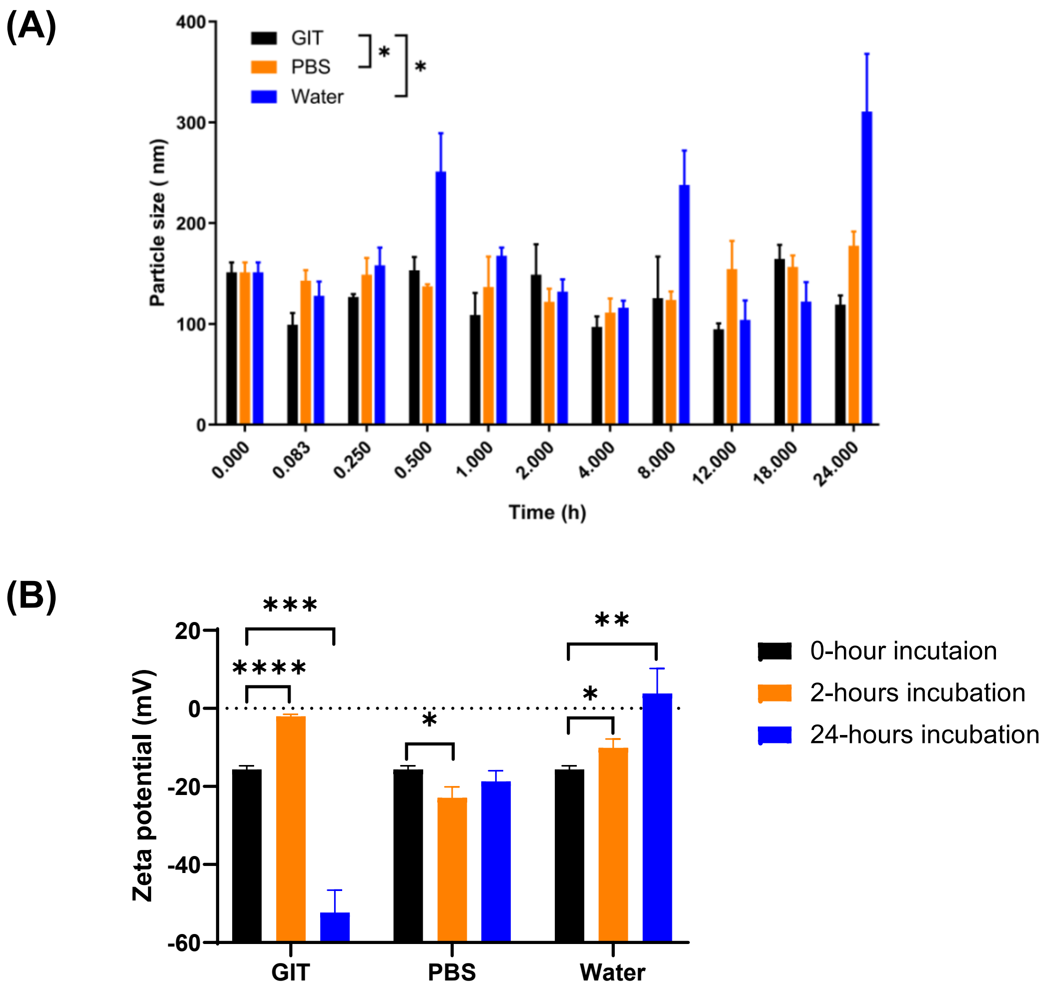

Figure S7: The *in vitro* stability of BENVs under physiological mimetic conditions. To evaluate the stability of BENVs in simulated gastrointestinal tract (GIT), BENVs were first incubated with simulated gastric fluid (pH 2.0) for 2 hours, then the solution was adjusted to pH 6.5 to mimic simulated intestinal fluid for the following 22 hours. The particle size **(A)** and zeta potential **(B)** of BENVs were measured using laser dynamic light scattering for 24 hours. $*p < 0.05$; $**p < 0.01$; $***p < 0.001$; $****p < 0.0001$ compared with their original values at 0 hour. Data shown are means ± S.D., n =3.

(A)

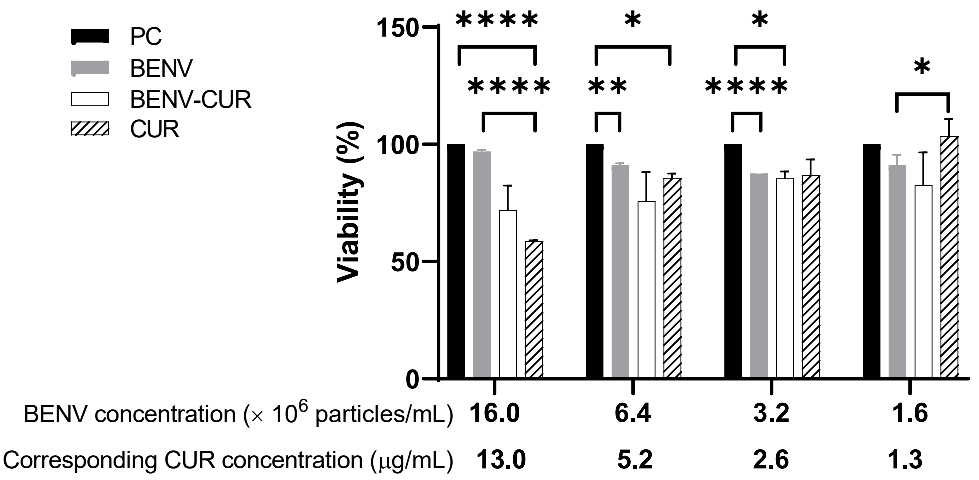

(B)

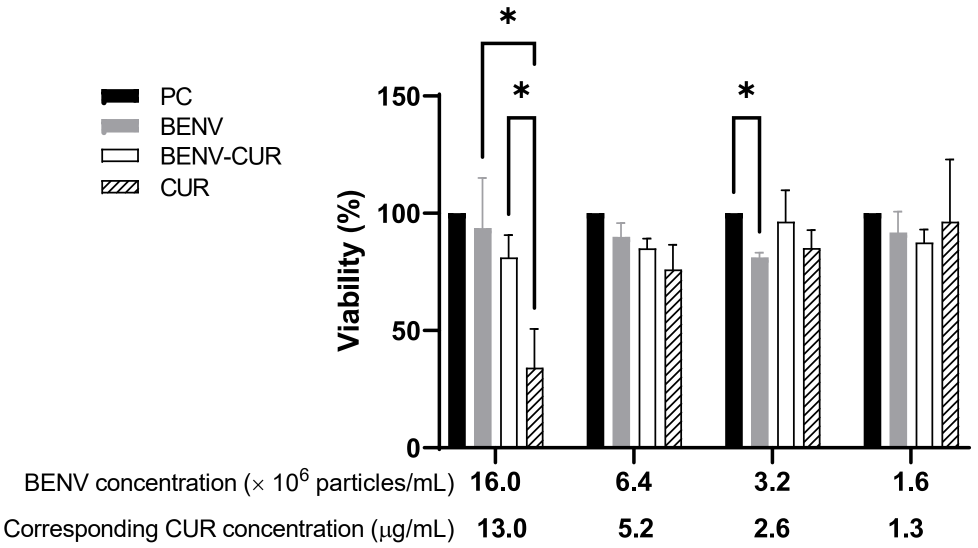

Figure S8. Cytotoxicity of BENV and curcumin-loaded BENV in HT-29 cells. Cell viability was determined for cells treated with free curcumin (CUR), blueberry derived extracellular nanoparticles (BENV), or curcumin-loaded BENV (BENV-CUR) for 48 hours (**A**) or 72 hours (**B**). The concentration of free CUR was equivalated to the CUR concentration encapsulated in BENVs. PC, non-treatment cells. CUR, curcumin. Data shown are means ± S.D., n=3. (*$p < 0.05$, **$p < 0.01$, ****$p < 0.0001$).

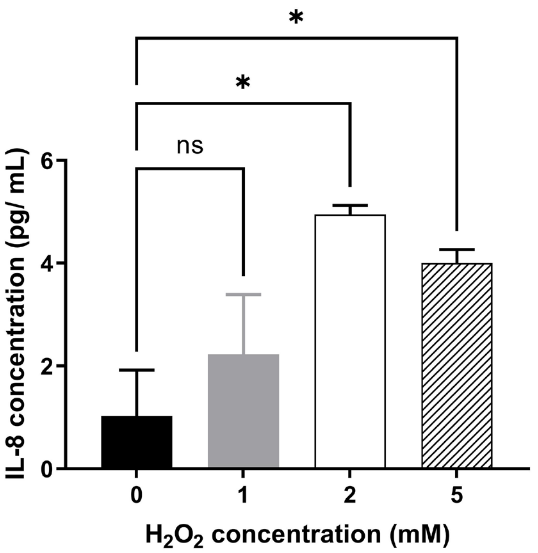

Figure S9. Effect of H_2O_2 concentration on IL-8 protein secretion in Caco-2 cells. Caco-2 cells were seeded 1 x 10^5 cells/well in a 24-well plate for 5 days, followed by the treatment of H_2O_2 at the indicated concentration for 6 hours. The IL-8 in cell culture medium was determined. NC, cells treated with vehicle only. Data shown are means ± S.D., n =3. (*$p < 0.05$).

Table S1. Comparison of the protein concentrations in EV samples isolated from different sources.

	Cell culture EVs	Milk EVs	AF	MJ 2k	MJ 10k	BENV 40k	BENV 100k
Protein concentration (mg/L)	0.5 - 2	335 ± 48	6772.93 ± 41.66	6760.20 ± 43.61	6760.65 ± 59.03	6174.04 ± 68.58	5599.96 ± 132.62

* Sample data were collected from cell culture-derived ENVs [3-5], milk-derived ENVs [6], apoplastic fluid (AF), minced juice collected at 2,000 × *g* (MJ 2k) and 10,000 × *g* (MJ 10k), BENVs isolated from AF at 40,000 × *g* (BENV 40k) and 100,000 × *g* (BENV 100k). Data are means ± S.D., n = 3.

References

1. Mihály, J., et al., *Characterization of extracellular vesicles by IR spectroscopy: Fast and simple classification based on amide and C H stretching vibrations.* Biochimica et Biophysica Acta (BBA)-Biomembranes, 2017. **1859**(3): p. 459-466.
2. Lätti, A.K., K.R. Riihinen, and P.S. Kainulainen, *Analysis of anthocyanin variation in wild populations of bilberry (Vaccinium myrtillus L.) in Finland.* Journal of agricultural and food chemistry, 2007. **56**(1): p. 190-196.
3. Sheng, H., et al., *Insulinoma-released exosomes or microparticles are immunostimulatory and can activate autoreactive T cells spontaneously developed in nonobese diabetic mice.* The Journal of Immunology, 2011. **187**(4): p. 1591-1600.
4. Estelles, A., et al., *Exosome nanovesicles displaying G protein-coupled receptors for drug discovery.* International journal of nanomedicine, 2007. **2**(4): p. 751.

5. Mitchell, J.P., et al., *Increased exosome production from tumour cell cultures using the Integra CELLine Culture System.* Journal of immunological methods, 2008. **335**(1-2): p. 98-105.
6. Munagala, R., et al., *Bovine milk-derived exosomes for drug delivery.* Cancer letters, 2016. **371**(1): p. 48-61.
